# Weight loss, insulin resistance, and study design confound results in a meta-analysis of animal models of fatty liver

**DOI:** 10.7554/eLife.56573

**Published:** 2020-10-16

**Authors:** Harriet Hunter, Dana de Gracia Hahn, Amedine Duret, Yu Ri Im, Qinrong Cheah, Jiawen Dong, Madison Fairey, Clarissa Hjalmarsson, Alice Li, Hong Kai Lim, Lorcan McKeown, Claudia-Gabriela Mitrofan, Raunak Rao, Mrudula Utukuri, Ian A Rowe, Jake P Mann

**Affiliations:** 1 School of Clinical Medicine, University of CambridgeCambridgeUnited Kingdom; 2 Leeds Institute for Medical Research & Leeds Institute for Data Analytics, University of LeedsLeedsUnited Kingdom; 3 Institute of Metabolic Science, University of CambridgeCambridgeUnited Kingdom; University of Texas Southwestern Medical CenterUnited States; McGill UniversityCanada

**Keywords:** fatty liver, meta-research, randomisation, bias, insulin resistance, Mouse, Rat

## Abstract

The classical drug development pipeline necessitates studies using animal models of human disease to gauge future efficacy in humans, however there is a low conversion rate from success in animals to humans. Non-alcoholic fatty liver disease (NAFLD) is a complex chronic disease without any established therapies and a major field of animal research. We performed a meta-analysis with meta-regression of 603 interventional rodent studies (10,364 animals) in NAFLD to assess which variables influenced treatment response. Weight loss and alleviation of insulin resistance were consistently associated with improvement in NAFLD. Multiple drug classes that do not affect weight in humans caused weight loss in animals. Other study design variables, such as age of animals and dietary composition, influenced the magnitude of treatment effect. Publication bias may have increased effect estimates by 37-79%. These findings help to explain the challenge of reproducibility and translation within the field of metabolism.

## eLife digest

Obesity and diabetes are increasingly common diseases that can lead to other complications such as fatty liver disease. Fatty liver disease affects one in five people and is caused by a built-up of fat in the liver, which can result in scarring of the liver tissue and other serious complications.

There is currently no cure for fatty liver disease. Drugs that have been effective in treating the condition in mice, lack efficacy in humans. To better understand why this is the case, Hunter, de Gracia Hahn, Duret, Im et al. conducted a review of over 5,000 published studies, analysing over 600 experiments.

Hunter et al. asked which drugs improved fatty liver in mice the most and if they had the same effect in humans. They also tested whether the age of the mice affected the outcome of the experiments. The analyses revealed that the drugs that work best in mice are different to the ones that show some effect in humans.

In mice, many of the drugs reduced their weight or lowered their blood sugar levels, which also improved the fatty liver condition. Moreover, drugs appeared to be less effective the older the mice were. However, most of these drugs do not cause weight loss or lower blood sugar levels in humans, suggesting that factors other than the intended action of these drug could affect the outcome of a mouse study.

These findings will help shape future research into obesity, diabetes and fatty liver disease using mice. They highlight that results obtained from studies with mice so far do not predict if a drug will work in humans to treat fatty liver disease. Moreover, weight loss seems to be the most important factor linked to how efficiently a drug treats fatty liver disease.

## Introduction

Interventional studies in animals are an integral component of drug development. If a disease can be suitably modelled in an animal, then the therapeutic response to a treatment observed in animals should inform its potential efficacy in humans (Howells et al., 2014). However, there is a well-documented translational gap between preclinical studies and subsequent outcomes in humans (Hackam and Redelmeier, 2006; Landis et al., 2012; Perel et al., 2007). Multiple factors contribute to this, including bias within study design (Macleod et al., 2015), insufficiently powered preclinical studies (Macleod et al., 2005), and biological differences between species (Mestas and Hughes, 2004; Rangarajan and Weinberg, 2003).

Systematic analyses of preclinical studies have found that publication bias may account for at least a third of the estimate of efficacy in trials (Henderson et al., 2015; Sena et al., 2010; van der Worp et al., 2010). In addition, other variables of animal model design can influence the magnitude of the treatment response (Watzlawick et al., 2019) and reporting of model design is often incomplete (Flórez-Vargas et al., 2016). These findings are highly relevant in the context of the ‘reproducibility crisis’ (Baker, 2016; von Herrath et al., 2019) as well as having ethical implications for the use of animals in research that is not of optimum quality (Prescott and Lidster, 2017).

Non-alcoholic fatty liver disease (NAFLD) is a highly active field of animal research (Brenner, 2018; Farrell et al., 2019). NAFLD is a common condition characterised by increased liver fat (hepatic steatosis) that may progress to inflammation in the form of non-alcoholic steatohepatitis (NASH) and fibrosis (Sanyal, 2019). Cirrhosis, end-stage liver disease, and hepatocellular carcinoma develop in a small proportion of patients. However, due to the high prevalence of obesity, NAFLD is the second most common indication for liver transplant in the United States (Younossi et al., 2018), predicted to overtake hepatitis C virus. NAFLD is intricately related with insulin resistance and therefore usually coexists with other features of the metabolic syndrome, such as type 2 diabetes and its recognised complications including cerebrovascular disease, coronary artery disease, and chronic kidney disease (Byrne and Targher, 2015).

There are currently no approved pharmacological therapies for NAFLD (Chalasani et al., 2018). Several Phase three trials are ongoing (Ratziu et al., 2019), but many interventions that appeared to have substantial efficacy in preclinical models have failed to be replicated in humans (Budas et al., 2016; Harrison et al., 2018; STELLAR-3 and STELLAR-4 Investigators et al., 2020; Sanyal et al., 2014). These studies have used a wide range of preclinical NAFLD models, including genetically modified animals (e.g. leptin deficient ob/ob mice), hypercaloric diets (e.g. high-fat diet), and toxic insults (e.g. streptozocin injections), all of which may be used in varying combinations and with different parameters (Anstee and Goldin, 2006). It is not known if, or which of, these variables influence treatment response to therapeutic agents in preclinical models of NAFLD, and which models are better predictors of response in humans.

Therefore, we performed a meta-analysis of interventional rodent studies of NAFLD to describe which drug classes were associated with improvement in NAFLD and whether any study characteristics (or biases) were linked to the magnitude of effect.

## Results

**Figure 1.**
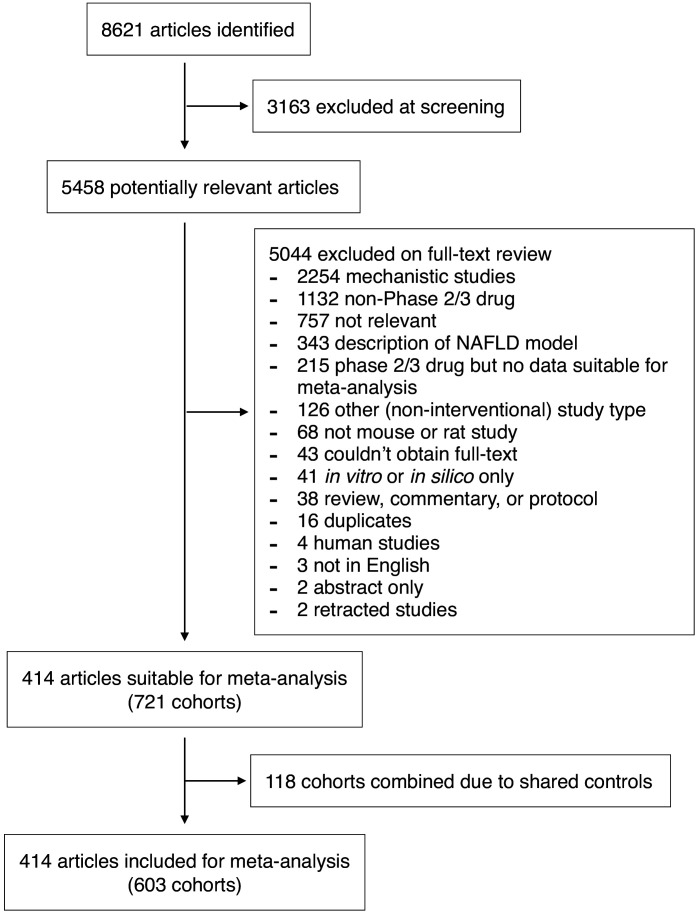
Study inclusion and exclusion flow chart. Figure 1—source data 1.Dataset used in this meta-analysis.Details and raw data of all studies included in the meta-analysis. This data can be used with the R code found in Supplementary Methods to run all analyses.

We performed a systematic search to identify interventional studies in rodent models of NAFLD. Our searches yielded 8621 articles, which after screening gave 5458 articles for full-text review (Figure 1). Studies were included in the meta-analysis if they used a pharmacological class that had been used in Phase 2 or three trials for NAFLD in humans (Supplementary file 1) and reported at least one of: hepatic triglyceride content, NAFLD Activity Score (NAS, or any of its components), portal inflammation, or fibrosis stage. After adjustments made for shared controls, 414 studies were included in the meta-analysis, comprising 603 cohorts of rodents (10,364 animals). Studies were predominantly performed in male animals (527/578, 91%). The median age at the start of intervention was 9-weeks old (range 0.6–80 weeks) for a median duration of 6 weeks (range 1 day – 60 weeks).

Hepatic triglyceride content was the most widely reported measure: 474/603 (79%) cohorts. Steatosis grade was the most frequently reported histological measure (174/603 (29%) cohorts), compared to: NAS 144/603 (24%), lobular inflammation 143/603 (24%), ballooning 106/603 (18%), and fibrosis in 58/603 (9.6%) cohorts. Portal inflammation was only reported in 8 cohorts from three studies, therefore meta-analysis was not possible for this outcome.

### Meta-analysis of hepatic triglyceride content

**Figure 2.**
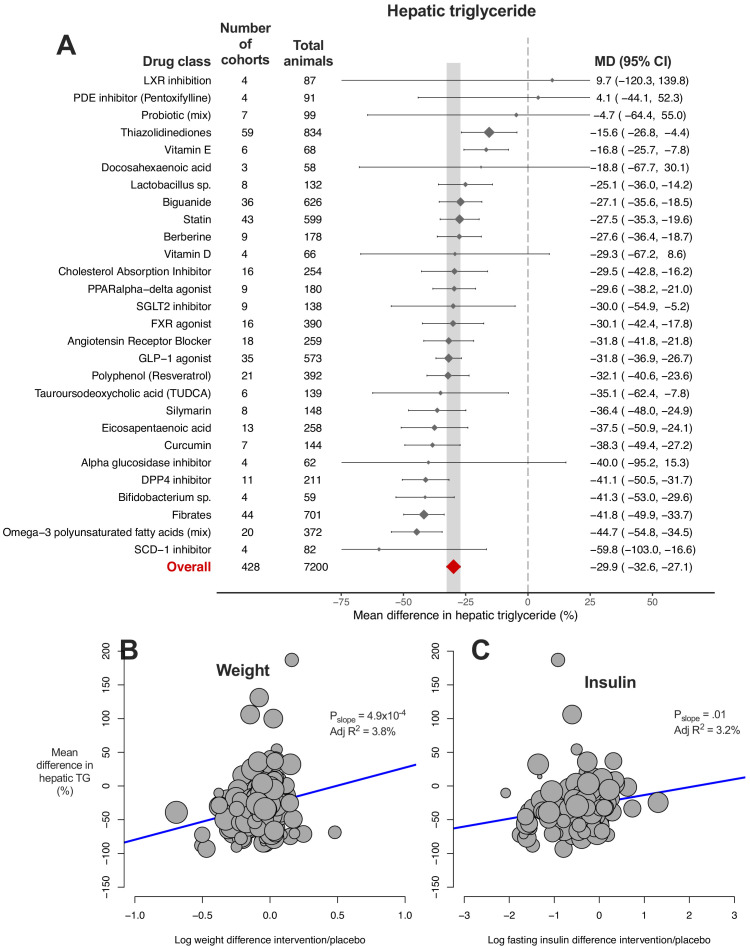
Meta-analysis of hepatic triglyceride content in rodent studies of NAFLD. (**A**) Forest plot with subgrouping by class of drug. Individual studies have been hidden and only subgroup summaries are illustrated. Results are expressed as a percentage difference relative to control (/placebo). The total number of animals per subgroup is calculated from the sum of control and interventional animals for each subgroup. CI, confidence interval; DPP4, Dipeptidyl peptidase-4; FXR, Farnesoid X receptor; GLP-1, Glucagon-like peptide-1; MD, mean difference; LXR, Liver X receptor; PDE, Phosphodiesterase; PPAR, Peroxisome proliferator-activated receptor; SCD-1, Stearoyl–CoA desaturase-1; SGLT2, Sodium-glucose co-transporter-2; TUDCA, Tauroursodeoxycholic acid. (**B**) Meta-regression bubble plot using (log) difference in weight between intervention and control animals, after removal of studies using models that induce weight loss. (**C**) Meta-regression bubble plot using (log) difference in fasting insulin between intervention and control animals, after removal of studies using models that induce weight loss. Figure 2—source data 1.Results of meta-analysis and meta-regression of hepatic triglyceride content in rodent studies of NAFLD.Tab 1. Results from meta-analysis of hepatic triglyceride with subgroup by drug class. Tab 2. Results from meta-analysis of hepatic triglyceride with subgroup by individual drug. Tab 3. Results from meta-analysis of hepatic triglyceride with subgroup by drug class, after removal of outlier studies. Tab 4. Results from univariable meta-regression analyses. Tab 5. Results from model 1 (without drug) and model 2 (including drug used) multivariable meta-regression analyses.

We used random-effects meta-analysis to estimate the mean difference (MD) in hepatic triglyceride (TG) content between intervention and control groups (Figure 2A). The overall mean difference in hepatic TG content was −29.9% (95% CI −33%, −27%) with considerable between-study heterogeneity (I^2^ = 90% (95% CI 89%, 90%), P_Q_ <1×10^−300^). Exclusion of outliers minimally affected the overall estimate (−30.2% (95% CI −33%, −27%), Figure 2—source data 1).

**Figure 2—figure supplement 1.**
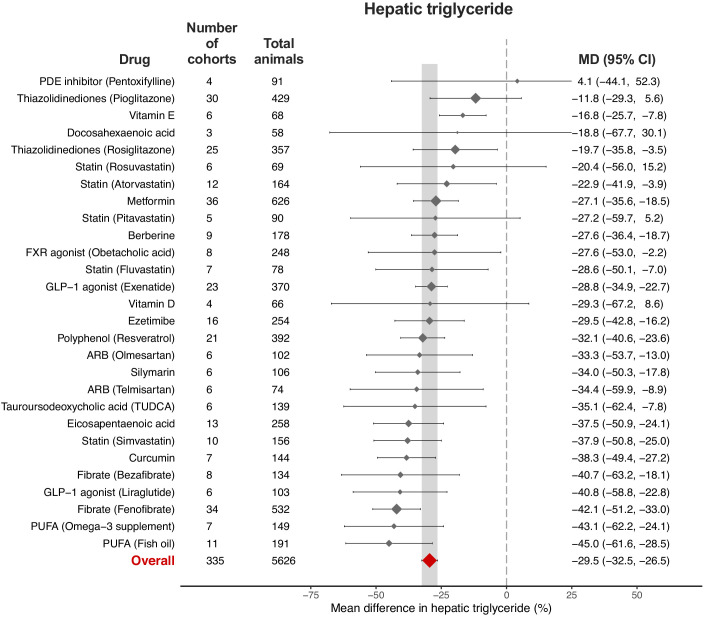
Meta-analysis of hepatic triglyceride content in rodent studies of NAFLD by individual drug. Forest plot with subgrouping by individual drug. Individual studies have been hidden and only subgroup summaries are illustrated. Results are expressed as a percentage change relative to control (/placebo). Total animals is the sum of control and interventional animals for each subgroup. ARB, angiotensin receptor blocker; CI, confidence interval; DPP4, Dipeptidyl peptidase-4; FXR, Farnesoid X receptor; GLP-1, Glucagon-like peptide-1; MD, mean difference; LXR, Liver X receptor; PDE, Phosphodiesterase; PPAR, Peroxisome proliferator-activated receptor; PUFA; omega-3 polyunsaturated fatty acid; SCD-1, Stearoyl–CoA desaturase-1; SGLT2, Sodium-glucose co-transporter-2; TUDCA, Tauroursodeoxycholic acid.

**Figure 2—figure supplement 2.**
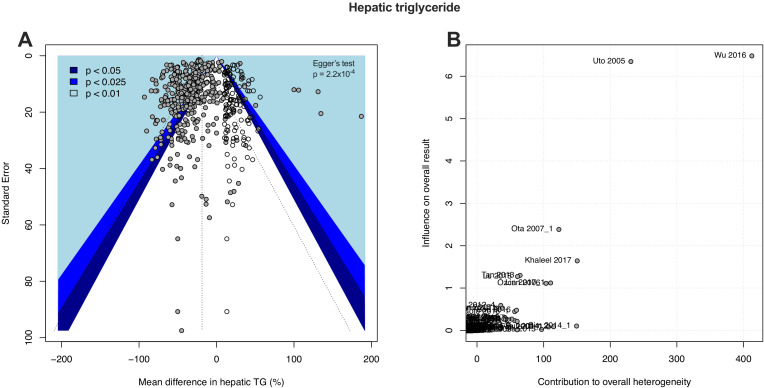
Funnel plot with trim-and-fill added studies and Baujat plot from meta-analysis of hepatic triglyceride content. (**A**) Funnel plot illustrating study distribution (publication) bias in 428 original studies (solid grey circles) with 125 added studies (from trim-and-fill). The statistical significance associated with each study is illustrated with the coloured background. Egger’s test p-value indicates the likelihood that the original studies came from a symmetrical distribution. (**B**) Baujat plot showing individual study contributions to heterogeneity in the meta-analysis. The studies with highest contribution were excluded in a sensitivity analysis.

For comparison, a relative decline of liver fat by ≥30%, as measured by magnetic resonance imaging proton-density fat fraction (MRI-PDFF), has been determined as the reduction required to achieve histological response in humans with NAFLD (Jayakumar et al., 2019; Loomba et al., 2020; Stine et al., 2020).

We hypothesised that much of this heterogeneity would be due to the different drug class interventions, with some classes having a greater effect than others. On meta-analysis using drug class as a subgroup, 22/28 (79%) of drug classes demonstrated a significant reduction in hepatic TG (i.e. the upper limit of their 95% CI was negative). If we were to use ≥30% reduction as a benchmark for clinical significance (analogous to change in MRI-PDFF), only 3/28 (11%) of drug classes passed this cut-off: fibrates, omega-3 polyunsaturated fatty acids (mixtures), and DPP-4 inhibitors.

The 95% CI of 24/28 drug classes overlapped with the CI of the overall effect estimate. Two drug classes, thiazolidinediones and vitamin E, were found to have a smaller mean reduction in hepatic TG and two classes had a greater reduction: fibrates and mixtures of omega-3 polyunsaturated fatty acids (PUFA). However, ‘PUFA mixtures’ was a comparatively broad drug class, and many PUFA mixtures included eicosapentaenoic acid (EPA) or docosahexaenoic acid (DHA), which individually showed no significant reduction in hepatic TG. There remained substantial or considerable heterogeneity within drug class subgroups (P_Q_ <0.05 for 21/28 drug classes, Figure 2—source data 1).

In order to investigate whether this heterogeneity was due to variation between individual drugs within classes we repeated the meta-analysis with subgroup by individual drugs (Figure 2—figure supplement 1). There was sufficient data for meta-analysis of 28 individual drugs (from the original 28 drug classes). 22/28 (79%) individual drugs were found to have a significant reduction in hepatic TG. Vitamin E was associated with a smaller mean reduction in hepatic TG than the 95% CI of the overall estimate, whilst fenofibrate was the only drug with a greater mean difference than the overall estimate. There remained considerable heterogeneity within subgroups for 20/28 drugs (I^2^ = 75–100%, P_Q_ <0.05).

We then performed univariable meta-regression to investigate which variables accounted for the heterogeneity in results (Figure 2—source data 1). Though individual drug used was the single variable that accounted for most heterogeneity (adj R^2^ = 4.9%, p=0.02), the majority of variation in results was unaccounted. An association was also observed for weight difference (adj R^2^ = 3.3%, p=6.4×10^−4^), where greater weight loss in the intervention group was associated with a greater reduction in hepatic TG. This association was stronger after removal of NAFLD models that induce weight loss (e.g. methionine-choline deficient diet (MCD), Figure 2B) and similar results were obtained for difference in fasting insulin levels (Figure 2C).

When these study characteristics were combined for multivariable meta-regression using an unbiased method, 10 variables were predicted to substantially contribute to the variation in hepatic TG difference (Table 1). In final model 1, weight difference was the only variable to be significantly associated with MD in hepatic TG (p=0.003). Including drug used in model two was able to account for all heterogeneity in results (Figure 2—source data 1) in a small subset of cohorts (k = 42), though neither of these models were significantly predictive of outcome following permutation tests (p-value*>0.05).

**Table 1.** Summary of findings across all outcomes and multivariable meta-regression analyses. Six separate meta-analyses were performed with subgrouping by classes of drug. Drug classes associated with outcome showed a significant reduction in the severity of NAFLD for that outcome, defined by the upper limit of their 95% confidence interval (CI). Differential efficacy refers to drug classes where their 95% CI did not overlap with that of the overall estimate. Multivariable meta-regression was performed using two models, where there was sufficient data: model one did not include drug class, model two included drug. For each analysis and model, the top variables are those identified to be substantially account for heterogeneity using multiple-variable inference. K refers to the number of cohorts included in each analysis. P-val* for each model refers to the overall model p-value (test of moderators) obtained after running multiple permutation tests, where p<0.1 should be considered indicative of an effect. ARB, angiotensin receptor blocker; DPP4-i, Dipeptidyl peptidase-4 inhibitor; EPA, eicosapentaenoic acid; FXR, Farnesoid X receptor; GLP-1, glucagon-like peptide-1; PPAR, peroxisome proliferator-activated receptor; PUFA; omega-3 polyunsaturated fatty acid; SCD1-i, stearoyl–CoA desaturase-1 inhibitor; SGLT2-i, sodium-glucose co-transporter-2 inhibitor; TUDCA, tauroursodeoxycholic acid.

	Meta-analysis with subgroup by drug class		Multi-variable meta-regression – model 1		Multi-variable meta-regression – model 2	
Outcome	Drug classes associated with outcome	Differential efficacy	Top predictors	Final model	Top predictors	Final model
Hepatic TG	22/28 (79%): SCD1-i, PUFA-mix, Fibrates, Bifidobacterium sp., DPP4-i, Curcumin, EPA, Silymarin, TUDCA, Polyphenol, GLP1 agonist, ARB, FXR agonist, SGLT2-i, PPARα-δ agonist, Cholesterol Absorption Inhibitor, Berberine, Statin, Biguanide, Lactobacillus sp., Vitamin E	Greater reduction: Fibrates, PUFA-mix Smaller reduction: Thiazolidinediones, Vitamin E	Weight, Insulin, Fat (%kcal), Model, Age at start, Background, Glucose, Sex, Duration, Quality score (k = 333)	R^2^ = 48.9%, P-val*=0.22 K = 67	Insulin, Fat (%kcal), Weight, Glucose, Age at start, Sex, Drug (k = 222)	R^2^ = 100%, P-val*=0.26 K = 42
Steatosis	9/22, (41%): Fibrates, GLP-1 agonist, DPP4-i, Probiotic (mix), Curcumin, Thiazolidinediones, Lactobacillus sp., Statin, ARB	Greater reduction: Fibrates	Glucose, Fat (%kcal), Sex (k = 94)	R^2^ = 91.8%, P-val*=0.03 K = 19	Fat (%kcal), Sex, Weight (k = 62)	R^2^ = 60.3%, P-val*=0.098 K = 27
Lobular inflammation	9/16 (56%): Fibrates, Probiotic (mix), Statin, ARB, FXR agonist, DPP4-i, Biguanide, Thiazolidinediones, Vitamin D	-	Glucose, Fat (%kcal) (k = 81)	R^2^ = 49.8%, P-val*=0.43 K = 19	-	-
Ballooning	8/14 (57%): Fibrates, Biguanide, Thiazolidinediones, Vitamin D, DPP4-i, ARB, FXR agonist, Probiotic (mix)	Greater reduction: Fibrates Smaller reduction: Probiotic (mix)	Glucose (k = 56)	R^2^ = 8.1%, P-val*=0.38 K = 26	-	-
NAFLD Activity Score	10/14 (71%):Fibrates, DPP4-i, GLP1 agonist, Probiotic (mix), Vitamin D, Silymarin, Biguanide, Thiazolidinediones, FXR agonist, ARB	Greater reduction: Fibrates	Glucose, Fat (%kcal), Age at start, Weight (k = 89)	R^2^ = 78.0%, P-val*=0.03 K = 19	Fat (%kcal), Weight, Background, Age at start, Sex (k = 58)	R^2^ = 63.1%, P-val*=0.001 K = 30
Fibrosis	2/5 (40%): FXR agonist, Statin	-	Model, Weight, Glucose, Fat (%kcal), Duration, Age at start (k = 58)	R^2^ = 100%, P-val*=0.67 K = 16	-	-

**Figure 3.**
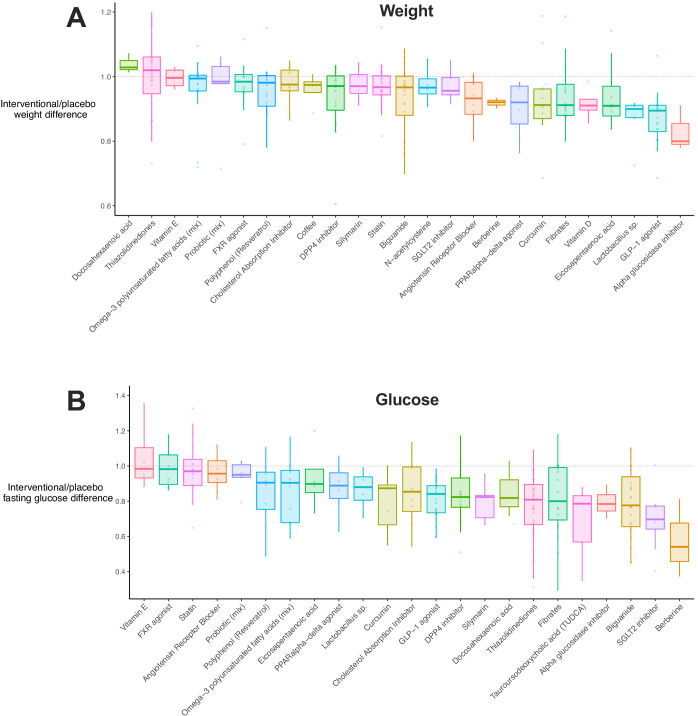
Weight and glucose difference associated with use of each drug class. (**A**) Box plot illustrating the difference in weight in interventional animals, expressed as a decimal of the weight of the control animals. Raw data points are plotted for each drug class. (**B**) Box plot for difference in fasting glucose in interventional animals, expressed as a decimal of the weight of the control animals. Raw data points are plotted for each drug class. Figure 3—source data 1.Results of difference in weight, glucose, and insulin for each drug class.Mean, standard deviation, and 95% confidence intervals for the percentage difference in weight, fasting glucose, and fasting insulin between interventional and placebo animals. ACC, acetyl-CoA carboxylase; ACE, angiotensin-2 converting enzyme; CB1, cannabinoid receptor 1; DPP4 Dipeptidyl peptidase-4; FXR, Farnesoid X receptor; GLP-1, glucagon-like peptide-1; LXR, liver X receptor; PDE, phosphodiesterase; PPAR, peroxisome proliferator-activated receptor; SCD1, stearoyl–CoA desaturase-1; SGLT2, sodium-glucose co-transporter-2; TUDCA, tauroursodeoxycholic acid; and UDCA, ursodeoxycholic acid.

Given that meta-regression implicated weight loss and improved insulin sensitivity in results, we explored how these traits were distributed by drug class (Figure 3A). Including all available data, we observed that 12/33 (36%) drug classes showed a significant reduction in weight (i.e. the upper limit of their 95% CI was below 1, Figure 3—source data 1). 17/32 (53%) and 15/25 (60%) of drug classes were associated with reductions in fasting glucose (Figure 3B) and insulin (Figure 3—figure supplement 1A), respectively. There was a positive correlation between weight, glucose, and insulin differences (Figure 3—figure supplement 1B). In addition, there was a negative correlation between weight difference and study duration or the age of mice at the end of intervention, that is longer studies (or those in older mice) were associated with greater weight loss in interventional groups.

**Figure 3—figure supplement 1.**
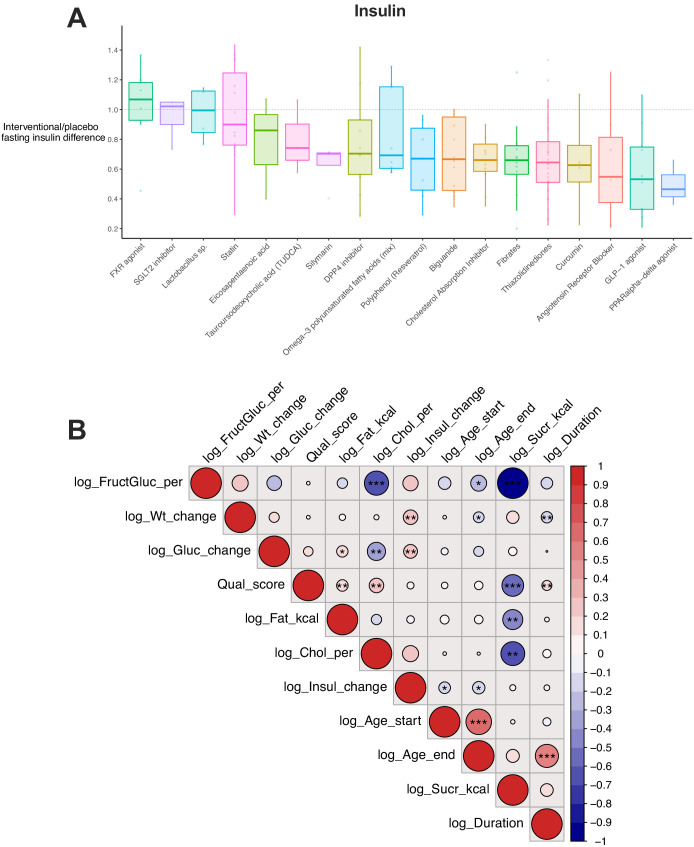
Insulin difference associated with use of each drug class and correlation plot of characteristics of studies. (**A**) Box plot illustrating the difference in fasting insulin in interventional animals, expressed as a decimal of the weight of the control animals. Raw data points are plotted for each drug class. (**B**) Plot of Pearson correlation co-efficients (encoded by colour, where red = 1, blue = −1) for continuous traits associated with each cohort. Traits have been log-transformed prior to analysis. Stars indicate p-value associated with each correlation: ***p<0.001, **p<0.01, *p<0.05.

We then explored whether these results showed study distribution (publication) bias or were heavily influenced by individual outliers (Figure 2—figure supplement 2). There was an uneven distribution of studies with a bias towards a reduction in hepatic TG, which was supported by Egger’s test (β = -.83 [95% CI −1.3, −0.4], p=2.2×10^−4^). Using the trim-and-fill method to account for this bias, we estimated that the true overall mean difference in hepatic TG would be −18.7% (95% CI −21%, −16%), over a third smaller than the original estimate.

### Meta-analysis of histological steatosis grade

**Figure 4.**
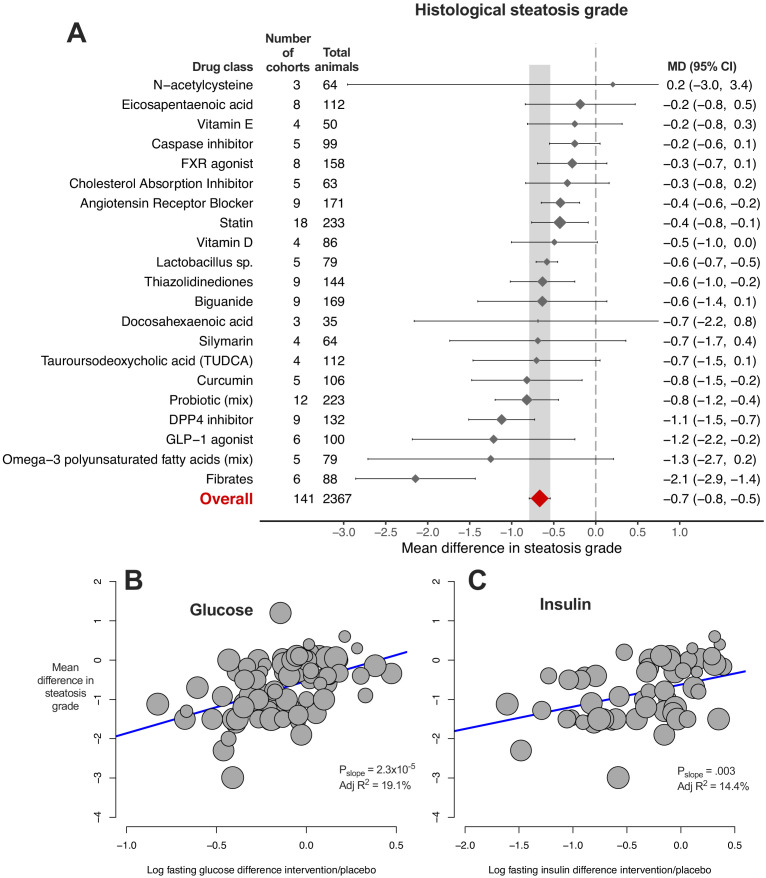
Meta-analysis of steatosis grade in rodent studies of NAFLD. (**A**) Forest plot with subgrouping by class of drug. Individual studies have been hidden and only subgroup summaries are illustrated. The total number of animals is calculated from the sum of control and interventional animals for each subgroup. CI, confidence interval; DPP4, Dipeptidyl peptidase-4; FXR, Farnesoid X receptor; GLP-1, Glucagon-like peptide-1; MD, mean difference; TUDCA, Tauroursodeoxycholic acid. (**B**) Meta-regression bubble plot using (log) difference in fasting glucose between interventional and control animals, after removal of studies using models that induce weight loss. (**C**) Meta-regression bubble plot using (log) difference in fasting insulin between interventional and control animals, after removal of studies using models that induce weight loss. Figure 4—source data 1.Results of meta-analysis and meta-regression of steatosis grade in rodent studies of NAFLD.Tab 1. Results from meta-analysis of steatosis grade with subgroup by drug class. Tab 2. Results from meta-analysis of steatosis grade with subgroup by individual drug. Tab 3. Results from meta-analysis of steatosis grade with subgroup by drug class, after removal of outlier studies. Tab 4. Results from univariable meta-regression analyses. Tab 5. Results from model 1 (without drug) and model 2 (including drug class used) multivariable meta-regression analyses.

Whilst hepatic TG was the most widely reported measure, histological assessment of disease is considered the gold standard for patients with NAFLD. Therefore, we performed a meta-analysis of MD in steatosis grade (Figure 4A). The overall MD in steatosis was −0.7 (95% CI −0.8, −0.5) again with considerable heterogeneity (I^2^ = 94% (95% CI 93%, 95%), P_Q_ <1×10^−300^). Compared to hepatic TG, fewer drug classes were identified to be associated with a significant reduction in steatosis grade (8/22, 36%), though again fibrates showed the largest effect size. Similar results were obtained when performing subgrouping by individual drugs, rather than classes (Figure 4—source data 1).

**Figure 4—figure supplement 1.**
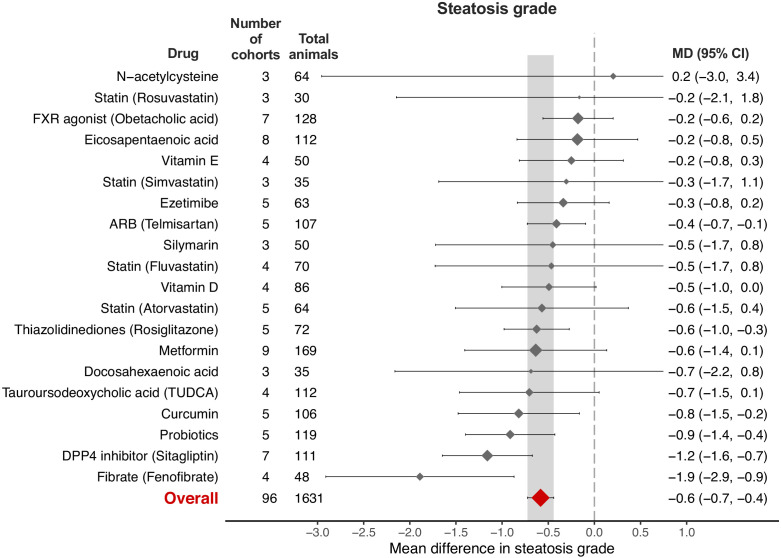
Meta-analysis of steatosis grade in rodent studies of NAFLD by individual drug. Forest plot with subgrouping by individual drug. Individual studies have been hidden and only subgroup summaries are illustrated. Total animals is the sum of control and interventional animals for each subgroup. ARB, angiotensin receptor blocker; CI, confidence interval; DPP4, Dipeptidyl peptidase-4; FXR, Farnesoid X receptor; MD, mean difference; TUDCA, tauroursodeoxycholic acid.

Univariable meta-regression found a marked association between difference in plasma glucose levels and MD in steatosis grade (Figure 4B, adj R^2^21%, p=2.4×10^−6^). Similar associations were observed for difference in weight and insulin levels, particularly after removal of weight-loss inducing models (Figure 4C). In addition, the sex of animals (adj R^2^7%, p=0.01) and genetic background were associated with MD in steatosis grade (Figure 4—source data 1). When factors were combined in multivariable meta-regression (Table 1), a model using sex, fasting glucose difference, and fat (%kcal) in diet accounted for 92% of variability in a small subset of cohorts (k = 19), which remained robust after a multiple permutation test (p-value*=0.03).

### Meta-analysis of lobular inflammation

**Figure 5.**
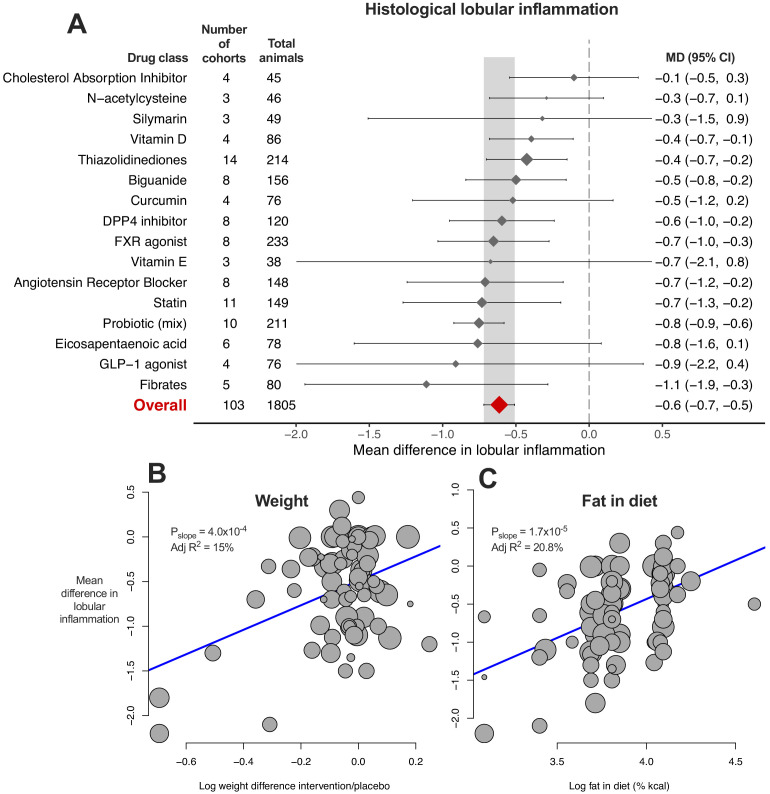
Meta-analysis of lobular inflammation in rodent studies of NAFLD. (**A**) Forest plot with subgrouping by class of drug. Individual studies have been hidden and only subgroup summaries are illustrated. The total number of animals is calculated from the sum of control and interventional animals for each subgroup. CI, confidence interval; DPP4, Dipeptidyl peptidase-4; FXR, Farnesoid X receptor; GLP-1, Glucagon-like peptide-1; MD, mean difference. (**B**) Meta-regression bubble plot using (log) difference in weight between interventional and control animals, after removal of studies using models that induce weight loss. (**C**) Meta-regression bubble plot using (log) fat (%kcal) in diet for each cohort. Figure 5—source data 1.Results of meta-analysis and meta-regression of lobular inflammation in rodent studies of NAFLD.Tab 1. Results from meta-analysis of lobular inflammation with subgroup by drug class. Tab 2. Results from meta-analysis of lobular inflammation with subgroup by individual drug. Tab 3. Results from meta-analysis of lobular inflammation with subgroup by drug class, after removal of outlier studies. Tab 4. Results from univariable meta-regression analyses. Tab 5. Results from multivariable meta-regression analyses.

9/16 (56%) drug classes were associated with a reduction in MD of lobular inflammation (Figure 5A). Again there was considerable heterogeneity within drug classes and when subgrouping by individual drugs (Figure 5—source data 1).

**Figure 5—figure supplement 1.**
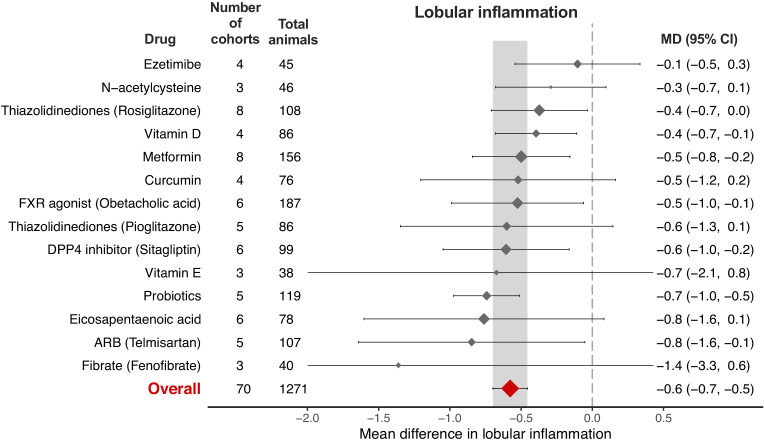
Meta-analysis of lobular inflammation in rodent studies of NAFLD by individual drug. Forest plot with subgrouping by individual drug. Individual studies have been hidden and only subgroup summaries are illustrated. Total animals is the sum of control and interventional animals for each subgroup. ARB, angiotensin receptor blocker; CI, confidence interval; DPP4, Dipeptidyl peptidase-4; FXR, Farnesoid X receptor; MD, mean difference.

Univariable meta-regression identified an association with difference in weight (Figure 5B, adj R^2^15%, p=4.0×10^−4^), as had been observed for steatosis grade and hepatic TG content. In addition, an association was found for fat %kcal in diet and MD in lobular inflammation: a higher %kcal fat in diet was associated with a smaller difference in lobular inflammation (Figure 5C, adj R^2^21%, p=1.7×10^−5^), indicating that study design was associated with size of treatment response. The bubble plot of fat content in diet also illustrated that the majority of studies reporting fat content in diet used either 40–45% or 60% kcal fat (Figure 5C).

### Meta-analysis of hepatocellular ballooning

**Figure 6.**
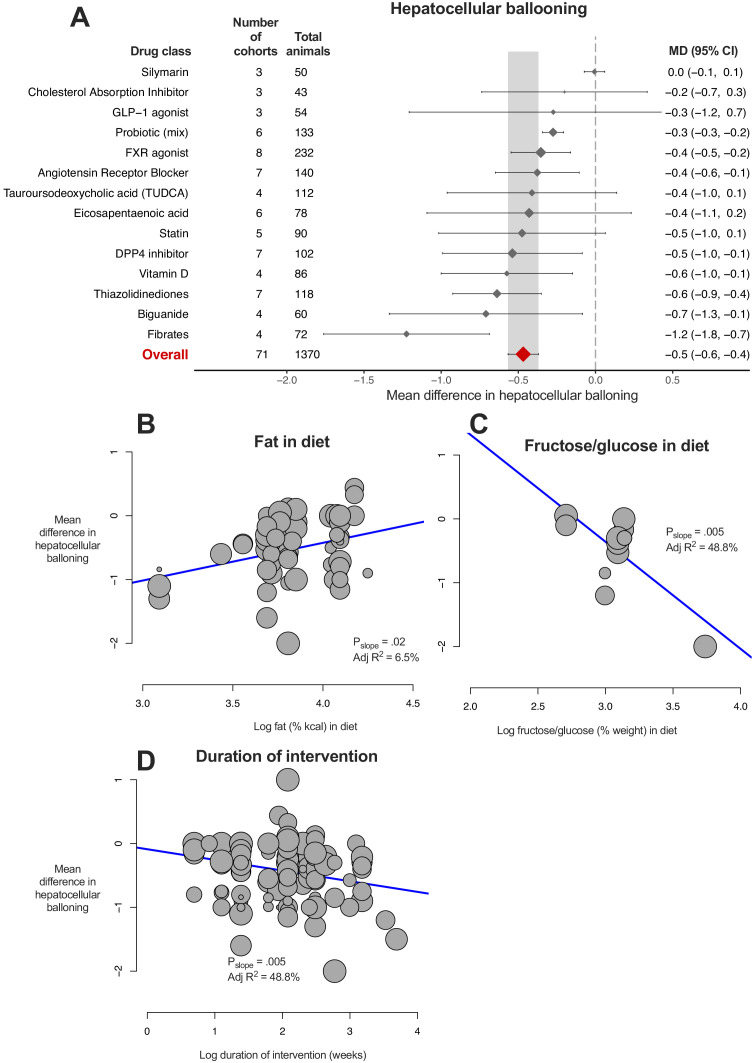
Meta-analysis of hepatocellular ballooning in rodent studies of NAFLD. (**A**) Forest plot with subgrouping by class of drug. Individual studies have been hidden and only subgroup summaries are illustrated. The total number of animals is calculated from the sum of control and interventional animals for each subgroup. CI, confidence interval; DPP4, Dipeptidyl peptidase-4; FXR, Farnesoid X receptor; GLP-1, Glucagon-like peptide-1; MD, mean difference; TUDCA, tauroursodeoxycholic acid. (**B**) Meta-regression bubble plot using (log) fat (%kcal) in diet for each cohort. (**C**) Meta-regression bubble plot using (log) fructose/glucose (% weight) in diet for each cohort. (**D**) Meta-regression bubble plot using (log) duration of intervention (in weeks) for each cohort. Figure 6—source data 1.Results of meta-analysis and meta-regression of hepatocellular ballooning in rodent studies of NAFLD.Tab 1. Results from meta-analysis of hepatocellular ballooning with subgroup by drug class. Tab 2. Results from meta-analysis of hepatocellular ballooning with subgroup by individual drug. Tab 3. Results from meta-analysis of hepatocellular ballooning with subgroup by drug class, after removal of outlier studies. Tab 4. Results from univariable meta-regression analyses.

8/14 (57%) drug classes were associated with a reduction in hepatocellular ballooning (Figure 6A). Fibrates showed greater reduction in ballooning than other studied drug classes, however this could not be replicated at an individual drug level (Figure 6—figure supplement 1).

**Figure 6—figure supplement 1.**
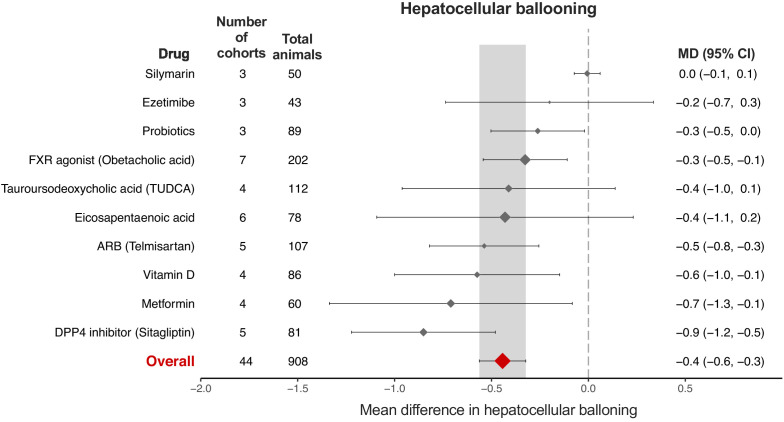
Meta-analysis of hepatocellular ballooning in rodent studies of NAFLD by individual drug. Forest plot with subgrouping by individual drug. Individual studies have been hidden and only subgroup summaries are illustrated. Total animals is the sum of control and interventional animals for each subgroup. ARB, angiotensin receptor blocker; CI, confidence interval; DPP4, Dipeptidyl peptidase-4; FXR, Farnesoid X receptor; MD, mean difference; TUDCA, tauroursodeoxycholic acid.

Similar to previous analyses, difference in fasting glucose (adj R^2^17%, p=9.0×10^−4^) and weight (adj R^2^8%, p=0.01) were associated with the magnitude of treatment effect. Study design characteristics also influenced difference in ballooning, namely percentage of fat in diet (Figure 6B, greater reduction in ballooning where a lower %kcal was used) and percentage of fructose/glucose in diet (Figure 6C); however, there were only 12 studies contributing to this analysis. In addition, longer studies were associated with larger reductions in ballooning severity (Figure 6D).

### Meta-analysis of NAFLD activity score (NAS)

**Figure 7.**
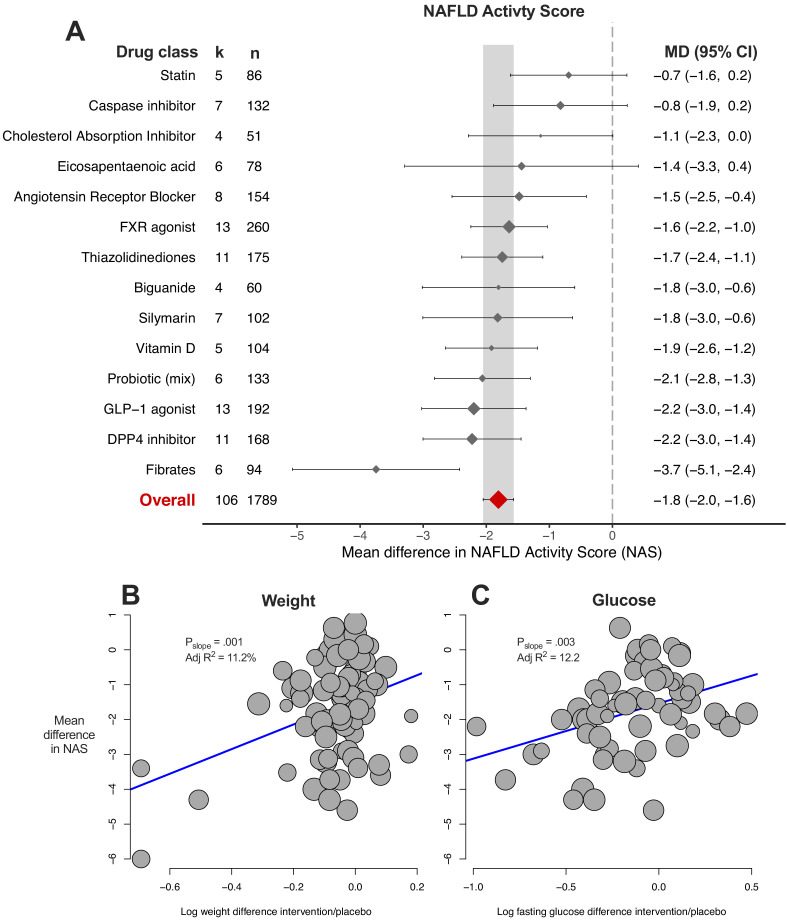
Meta-analysis of NAFLD Activity Score (NAS) in rodent studies of NAFLD. (**A**) Forest plot with subgrouping by class of drug. Individual studies have been hidden and only subgroup summaries are illustrated. k represents the number of cohorts in each subgroup. The total number of animals is calculated from the sum of control and interventional animals for each subgroup. CI, confidence interval; DPP4, Dipeptidyl peptidase-4; FXR, Farnesoid X receptor; GLP-1, Glucagon-like peptide-1; MD, mean difference. (**B**) Meta-regression bubble plot using (log) difference in weight between interventional and control animals, after removal of studies using models that induce weight loss. (**C**) Meta-regression bubble plot using (log) difference in glucose between interventional and control animals, after removal of studies using models that induce weight loss. Figure 7—source data 1.Results of meta-analysis and meta-regression of NAFLD Activity Score (NAS) in rodent studies of NAFLD.Tab 1. Results from meta-analysis of NAS with subgroup by drug class. Tab 2. Results from meta-analysis of NAS with subgroup by individual drug. Tab 3. Results from meta-analysis of NAS with subgroup by drug class, after removal of outlier studies. Tab 4. Results from univariable meta-regression analyses. Tab 5. Results from model 1 (without drug) and model 2 (including drug used) multivariable meta-regression analyses.

The NAFLD activity score is a composite of steatosis, lobular inflammation, and ballooning scores. The results largely reflected those observed for the previous three meta-analyses (Figure 7A). 10/14 (71%) drug classes were associated with a significant reduction in NAS, with fibrates being the most beneficial drug class. Meta-regression found associations for difference in weight (Figure 7B) and glucose (Figure 7C) to account for 11% and 12% of heterogeneity in results, respectively.

**Figure 7—figure supplement 1.**
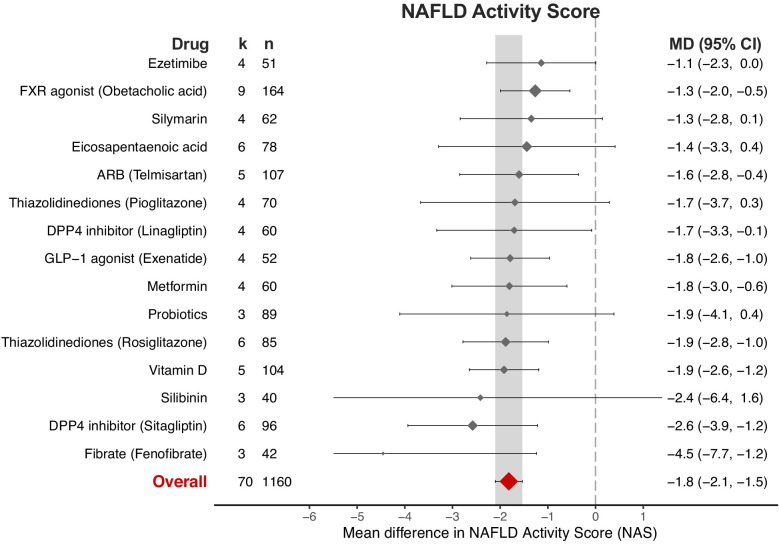
Meta-analysis of NAFLD Activity Score (NAS) in rodent studies of NAFLD by individual drug. Forest plot with subgrouping by individual drug. Individual studies have been hidden and only subgroup summaries are illustrated. k represents the number of cohorts in each subgroup. Total animals is the sum of control and interventional animals for each subgroup. ARB, angiotensin receptor blocker; CI, confidence interval; DPP4, Dipeptidyl peptidase-4; FXR, Farnesoid X receptor; MD, mean difference.

multiple-variable meta-regression models were able to account for more than 60% of variation in results (in a small subset of cohorts) using genetic background, fat in diet, age at start of intervention, weight and glucose difference, but without requiring drug or drug class (Table 1).

### Meta-analysis of fibrosis stage

**Figure 8.**
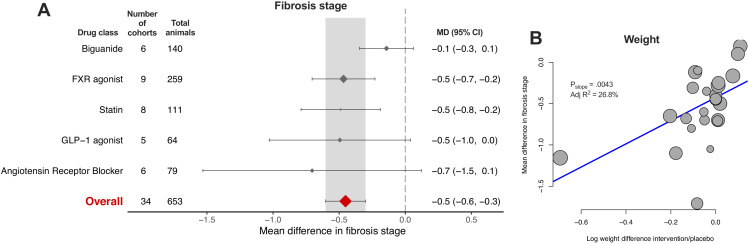
Meta-analysis of fibrosis stage in rodent studies of NAFLD. (**A**) Forest plot with subgrouping by class of drug. Individual studies have been hidden and only subgroup summaries are illustrated. The total number of animals is calculated from the sum of control and interventional animals for each subgroup. CI, confidence interval; FXR, Farnesoid X receptor; GLP-1, Glucagon-like peptide-1; MD, mean difference. (**B**) Meta-regression bubble plot using (log) difference in weight between interventional and control animals, after removal of studies using models that induce weight loss. Figure 8—source data 1.Results of meta-analysis and meta-regression of fibrosis stage in rodent studies of NAFLD.Tab 1. Results from meta-analysis of fibrosis stage with subgroup by drug class. Tab 2. Results from meta-analysis of fibrosis stage with subgroup by individual drug. Tab 3. Results from meta-analysis of fibrosis stage with subgroup by drug class, after removal of outlier studies. Tab 4. Results from univariable meta-regression analyses. Tab 5. Results from multivariable meta-regression analyses.

Fibrosis stage is the histological feature that most strongly correlates with liver-related outcomes in humans with NAFLD (Angulo et al., 2015; Ekstedt et al., 2015), and was therefore pre-specified as the primary outcome measure for this study. However, it was reported in only 58/603 (9.6%) of cohorts. Only FXR agonists and statins (2/5, 40% drug classes) were associated with a significant reduction in fibrosis stage (Figure 8A), where the overall mean difference was −0.5 (95% CI −0.6, −0.3) stages. Meta-regression replicated previous findings for other traits, showing that difference in weight was associated with reduction in fibrosis stage (Figure 8B, adj R^2^27%, p=0.004).

**Figure 8—figure supplement 1.**
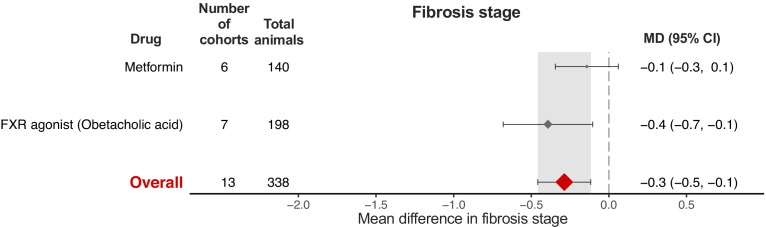
Meta-analysis of fibrosis stage in rodent studies of NAFLD by individual drug. Forest plot with subgrouping by individual drug. Individual studies have been hidden and only subgroup summaries are illustrated. Total animals is the sum of control and interventional animals for each subgroup. CI, confidence interval; FXR, Farnesoid X receptor; MD, mean difference.

### Bias analyses of histological outcomes and study quality

**Figure 9.**
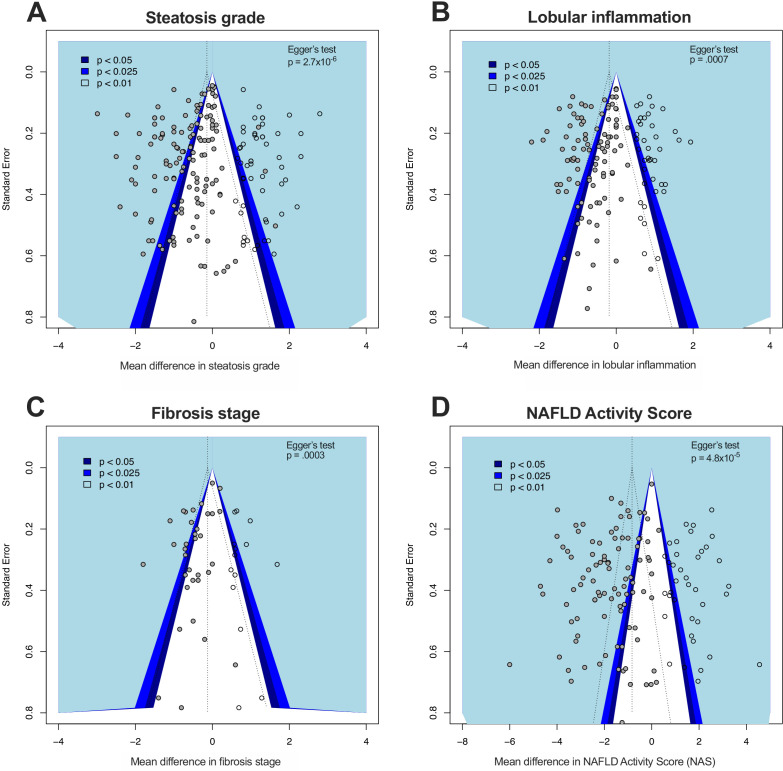
Funnel plots illustrating study distribution bias from meta-analyses of histological features. (**A**) Funnel plot illustrating study distribution (publication) bias in 145 original studies (solid grey circles) with 54 added studies (from trim-and-fill) for meta-analysis of steatosis grade. The statistical significance associated with each study is illustrated with the coloured background. Egger’s test p-value indicates the likelihood that the original studies came from a symmetrical distribution. (**B**) Funnel plot for lobular inflammation meta-analysis with 103 original studies and 42 added studies. (**C**) Funnel plot for fibrosis stage meta-analysis with 34 original studies and 14 added studies. (**D**) Funnel plot for NAS meta-analysis with 106 original studies and 43 added studies.

Funnel plots for steatosis grade, lobular inflammation, fibrosis stage, and NAS were asymmetric (Figure 9), supported by the results of Egger’s test for each analysis.

**Figure 9—figure supplement 1.**
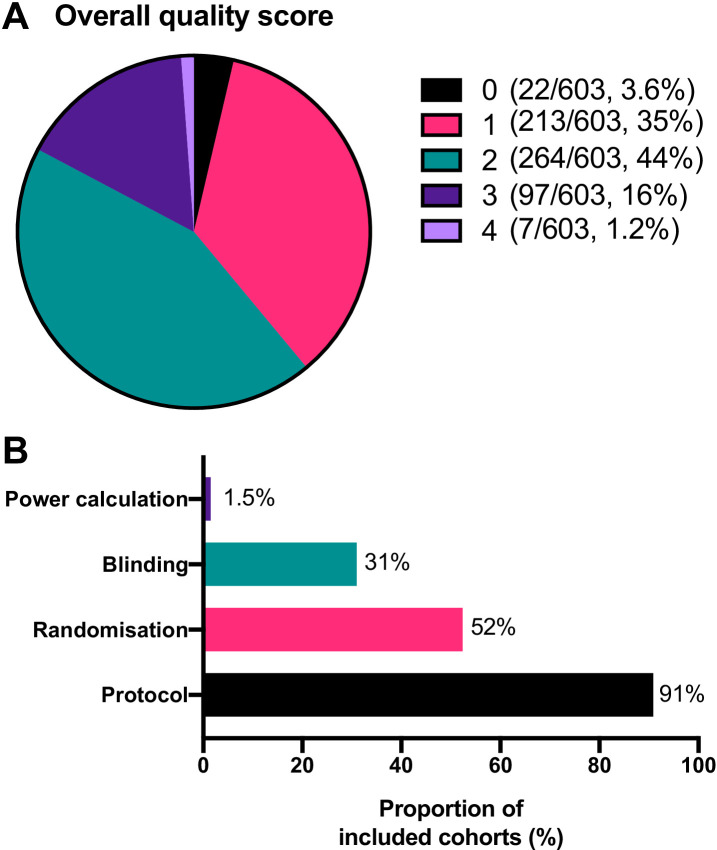
Quality assessment of included cohorts. (**A**) Distribution of overall quality scores from a four-point scale, composed of the use of a power calculation, use of blinding, randomisation, and referring to a predefined protocol, with 1-point awarded for presence of each factor. (**B**) Proportion of cohorts achieving each factor is shown in B.

Using the trim-and-fill method to account for these differences substantially altered the overall effect estimates: for steatosis grade, there was an 79% reduction in estimated effect size to −0.14 (95% −0.3, +.01); for lobular inflammation, a 70% reduction in effect size to −0.18 (95% −0.32, −0.05); for fibrosis, 72% reduction to −0.12 (95% −0.33, +.08); and NAS, 55% reduction in effect size to −0.82 (95% −0.1.1, −0.5).

We used a four-item scale to estimate study quality (Figure 9—figure supplement 1). We found that 497/603 (82%) cohorts were at high risk of bias due to either absence of randomisation or absence of blinding. In addition, we used post-hoc power calculations to estimate the proportion of studies that were adequately powered. For analysis of hepatic TG, 39% (185/474) cohorts had a power of 80% or greater on post-hoc calculation. However, using the results from this meta-analysis, to achieve a power of 80% with significance set as p=0.05, group size would need to be n = 16. 4.2% (20/474) cohorts included 16 or more animals and would have met sufficient power to detect associations, based on these data.

Similar results were obtained for histological steatosis grade: 70/174 (40%) reported results consistent with >80% power but only 27/174 (16%) had a group size large enough to be expected to reach 80% power.

### Summary of findings across traits

The majority of drug classes (or individual drugs) were found to show a significant reduction in severity of NAFLD. Fibrates (for which most data were available for fenofibrate) demonstrated the greatest improvement in several outcome measures (Table 1).

**Figure 10.**
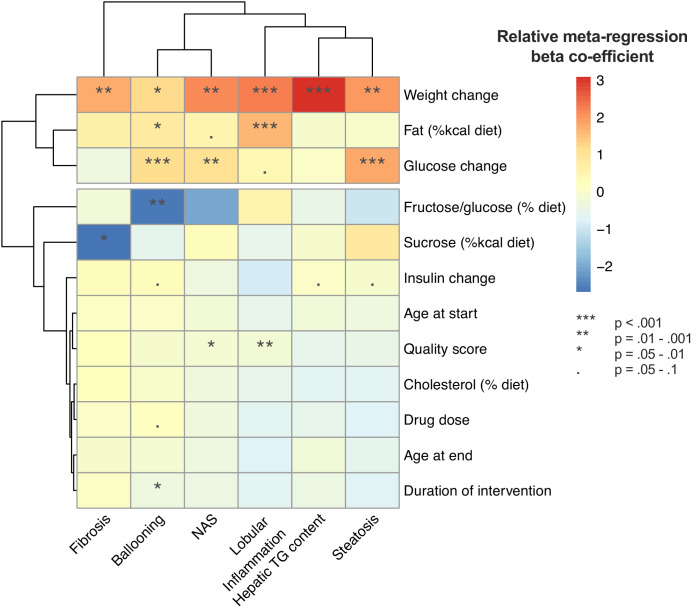
Summary of univariable meta-regression results across all outcomes.

Univariable meta-regression found that weight loss and lower fasting glucose were associated with a greater improvement in multiple outcomes (Figure 10). In addition, diet composition influenced the magnitude of treatment response for lobular inflammation, ballooning, and fibrosis.

Heatmap illustrating the results of univariable meta-regression analyses using continuous variables. Beta-regression co-efficient was normalized within each outcome analysis (e.g. steatosis grade) to mean = 0, standard deviation = 1. Rows (variables used as predictors in meta-regression) and columns (outcome measures for NAFLD) are clustered for similarity.

## Discussion

Through meta-analysis and meta-regression we have illustrated that weight loss and alleviation of insulin resistance are consistently associated with treatment response in interventional trials for NAFLD in rodents. This extends beyond drugs that cause weight loss in humans. In addition, we have found that study design characteristics (e.g. diet composition) can influence the magnitude of treatment response. These findings suggest that factors other than the pharmacological mechanism of the trialled drug may confound the results observed in such studies.

All stages of NAFLD show a strong, positive correlation with severity of insulin resistance in humans and type 2 diabetes is a major risk factor for the presence of advanced fibrosis (Younossi et al., 2019). Consistent with this, weight loss and improvement in insulin sensitivity are associated with histological improvement in NAFLD (Koutoukidis et al., 2019), particularly evident from studies of bariatric surgery (Lassailly et al., 2015; Lee et al., 2019) and liraglutide (Armstrong et al., 2015). Therefore, it is not a surprising observation to see this replicated in our meta-regression analyses and it is consistent with previous observations (Hui et al., 2015). On multiple-variable inference, weight loss or fasting glucose were the most important variables across several outcome metrics. This provides strong evidence that (in rodents) alleviation of insulin resistance, usually mediated by weight loss, improves features of NAFLD, independent of the drug used.

Some drug classes that caused weight loss in rodents are also well established to cause weight loss in humans (e.g. GLP-1 agonists and metformin), whilst others are not (e.g. vitamin D and statins). The findings for insulin sensitivity were similar, with over 50% of drugs reducing fasting glucose. Again, some drugs were consistent with their effect in humans (e.g. thiazolidinediones, DPP4-inhibitors) but not others (e.g. ezetimibe). It is not clear whether this is due to reduced food intake or other toxic effects of the drugs. It should be noted that some individual studies faithfully recapitulated observations in humans, for example weight gain, adipose expansion, and improved insulin sensitivity with thiazolidinedione use. However across the dataset as a whole, these observations suggest that ‘off-pharmacological-target’ effects, causing changes in weight and glucose homeostasis, may account for some of the translational gap between agents efficacious in rodents but not humans.

Though there are no licensed therapies for NAFLD, drug development is a highly active field (Friedman et al., 2018) and there have been over 30 drugs used in Phase 2 or three trials. Some have demonstrated potential efficacy in well-conducted randomized controlled trials, most notably GLP-1 agonists (Armstrong et al., 2015) and pioglitazone (Cusi et al., 2016; Sanyal et al., 2010). However, the majority of early phase trials did not find substantial benefit from the trialled interventions (Supplementary file 1). Whereas in animals, a large number of drugs (and classes) demonstrated significant efficacy across several outcome measures. This did not appear to be consistent with the results from human trials, for example we observed that vitamin D was associated with a significant reduction in NAS, however several trials have not found any benefit from its use in humans (Barchetta et al., 2016; Dabbaghmanesh et al., 2018). In addition, the magnitude of effect observed in rodents was not consistent with human data. For example, there is reasonably convincing evidence that pioglitazone improves NAFLD in humans, however it had one of the smallest improvements in hepatic TG. Similarly, GLP-1 agonists, which met their primary outcome in a human Phase two study (Armstrong et al., 2015), rank in the middle for most outcomes in this analysis. Fibrates had one of the largest treatment effects across multiple analyses but this does not appear to be consistent with human evidence to date (Fabbrini et al., 2010; Oscarsson et al., 2018). Fibrate use was also associated with a median 10% wt loss in these analyses, which has not been observed in large randomised trials in humans (Keech et al., 2005). Even though we found evidence for efficacy of the majority of drugs included in this analysis, the 95% CI for treatment effect size overlapped for most drug classes. This is generally consistent with findings reported in preclinical models of spinal cord injury where the effect size of several different types of treatment overlapped (Watzlawick et al., 2019). Overall, the trends observed are not consistent with findings in humans and there does not appear to be any clear patterns that indicate potentially successful translation.

Several study design characteristics affected treatment response across multiple outcome measures, including the age of animals, sex, genetic background, and dietary composition. There are a huge number of variables in the design of an interventional animal study and many were simplified for the input into analyses. For example, the ‘model’ used was simplified to a ‘core’ model (e.g. leptin deficient (ob/ob) mice) and separated from the genetic background of the animals for this analysis. Similarly, we studied several dietary components in isolation, which could have led to the observation that a higher proportion of dietary fat (e.g. 60% kcal) was associated with a smaller treatment response. This may be because lower fat containing diets (e.g. 40% kcal) may be combined with added cholesterol or other components, such as fructose. However these data do illustrate the concept that multiple factors associated with model design influence not only animal phenotype but magnitude of treatment response. This was demonstrated using multiple-variable meta-regression models where in some analyses the majority of variation in results could be accounted for (in a small subset of cohorts) without including drug as a covariate, particularly for NAS and steatosis grade.

It should be noted that there have been more systematic analyses of genetic background on NAFLD (Chella Krishnan et al., 2018; Hui et al., 2015) as well as in other fields, including immunology (Martin et al., 2017) and behavioural neuroscience (Homanics et al., 1999; Liu and Gershenfeld, 2001). We were surprised to find that genetic background was a top variable in comparatively few of our multivariate models. Based on observations from the Hybrid Mouse Diversity Panel (Chella Krishnan et al., 2018; Hui et al., 2018; Hui et al., 2015), we anticipate that the true impact of genetic background may be greater than we could quantify, due to our inclusion of a narrow range of backgrounds that had been used in multiple studies and our exclusion of mixed genetic backgrounds from analysis.

The vast majority of included studies demonstrated an improvement in NAFLD, which could be partly accounted for by a trend towards reporting positive results that is publication bias. Using the trim-and-fill method, we estimated that study distribution bias (most likely publication bias in this case) may have substantially increased the reported magnitude of effect (e.g. overall reduction in hepatic TG of 19% compared to 30%). The presence of publication bias did not come as a surprise (Tsilidis et al., 2013) and this dataset provides useful replication of the strong evidence base for this in preclinical neurological studies. A previous work on preclinical models of sunitinib calculated the overestimate from potential publication bias at 45% (Henderson et al., 2015). The results from power calculations are also likely to reflect publication bias: based on the overall effect summary, a minority of cohorts were of sufficient size to be predicted to achieve the power of 80%. Similarly, we have replicated previously described low rates of randomisation and blinding in animal studies (Bahor et al., 2017).

We found very few studies to report portal inflammation severity. In humans, (peri-)portal inflammatory activity has been shown to correlate with severity of fibrosis in both adults and children with NAFLD (Brunt et al., 2009; Mann et al., 2016; Rakha et al., 2010). Therefore, this remains a relatively unexplored area worthy of investigation, as targeting portal inflammation may be beneficial in slowing disease progression.

There are several implications of these results. Firstly, it is not surprising that there are multiple reports of difficulty in reproducing preclinical studies in the field of metabolism (von Herrath et al., 2019) given that study design has a considerable effect on treatment response. Variations in what may appear to be small details (such as age at the start of study diet) influence results and therefore could silence subtle differences or generate false positives.

Secondly, these results also help to explain the difficulty in bridging the preclinical to human translational gap (Denayer et al., 2014), which might be relevant beyond the field of metabolism research. For example, we did not observe an association between drug dose and treatment effect size. In addition, studies were overwhelmingly performed in male animals, whereas human studies are more evenly balanced (e.g. 60% female in the ‘STELLAR-3/–4’ trial [STELLAR-3 and STELLAR-4 Investigators et al., 2020]). Sex was a top predictor of several multivariable inference models and therefore the lack of inclusion of female mice may hinder identification of drugs for translation. Similarly, studies were almost uniformly done on young mice who were growing, unlike the focus on adult patients in all major phase 3 NAFLD trials.

The main strength of this work is the number of included studies, interventions, and variables. This has facilitated a detailed analysis of a single disease area. However this study has simplified some study characteristics to facilitate meta-regression analyses, which may have under-estimated the impact of particular variables on outcome measures. One such simplification was grouping of drugs into classes, some of which (e.g. ‘Probiotics (mix)’) were comparatively vague, compared to those with well-defined mechanisms (e.g. thiazolidinediones). Similarly, we used a simplified categorisation of rodent models (e.g. high-fat diet), combined with individual continuous metrics (e.g. fat %kcal), which will not capture the full variation of models used. We used fasting glucose and insulin as proxies for insulin resistance, however these are not direct measures of insulin resistance. This would require results from hyperinsulinaemic-euglycaemic clamps, or at least insulin tolerance tests, but these were performed in comparatively few studies. Similarly, we elected to record histological outcomes only where it was reported according to standard criteria for reporting human biopsies of NAFLD. There are a wide variety of other methods of interpreting liver histology, some of which are more quantitative (e.g. collagen proportionate area), though again these were less frequently reported. It should also be noted that this study did not have a pre-specified statistical analysis plan, which increases its risk of bias.

There is a wide range of other variables that were not considered in this analysis. Some were unreported variables, such as technique of animal handling. A further factor of potential relevance is the bacterial status of rodents, which is known to affect liver phenotypes (Kaden-Volynets et al., 2019), potentially via intestinal dysbiosis (Balmer et al., 2014; Mazagova et al., 2015). Furthermore, many studies did not report certain variables, for example genetic background of animals was not reported in 5.3% (32/603), which reduced the number of studies included in meta-regression analyses. This was most obvious for multiple-variable meta-regression, where some final models included fewer that 20 data points. However this meta-analysis has included a large number of articles, which gives considerable confidence in the findings we have replicated across several outcome measures.

### Conclusion

Multiple drug classes improve NAFLD in rodents, however these results may be confounded by weight loss and alleviation of insulin resistance not observed in humans treated with the same drugs. Publication bias over-estimates these effect sizes by at least a third and a variety of other study design characteristics also influence treatment response. Therefore, standardisation of practices is needed in preclinical studies of metabolism to improve the translatability and reproducibility of findings.

## Materials and methods

**Key resources table:** 

Reagent type (species) or resource	Designation	Source or reference	Identifiers	Additional information
Software, algorithm	R [base], dmetar (RRID:SCR_019054), metaphor (RRID:SCR_003450), meta (RRID:SCR_019055)	R	R 4.0.2	
Software, algorithm	GraphPad Prism (RRID:SCR_002798)	GraphPad Prism	GraphPad Prism v8	

### Review protocol and search strategy

The systematic review protocol was prospectively registered with SyRF (Systematic Review Facility) and is available from: https://drive.google.com/file/d/0B7Z0eAxKc8ApQ0p4OG5SblRlRTA/view.

PubMed via MEDLINE and EMBASE was searched for published articles of experimental rodent models of fatty liver, NAFLD, or non-alcoholic steatohepatitis (NASH). The following search term was used: (‘Non-alcoholic fatty liver disease’ OR ‘Nonalcoholic fatty liver disease’ OR ‘NAFLD’ OR ‘non-alcoholic steatohepatitis’ OR ‘nonalcoholic steatohepatitis’ OR ‘NASH’ OR ‘fatty liver’ OR ‘hepatic steatosis’) AND (‘mouse’ OR ‘animal’ OR ‘rat’ OR ‘murine’ OR ‘animal model’ OR ‘murine model’ OR ‘rodent model’ OR ‘experimental model’) NOT (‘Review’). Both databases were searched using the ‘Animal’ filters (de Vries et al., 2014; Hooijmans et al., 2010), the results combined, and duplicates eliminated. The search was completed in January 2019.

### Study selection and eligibility criteria

Our inclusion criteria were as follows: primary research articles using mice or rats to model NAFLD (to include hepatic steatosis, NASH, and NASH-fibrosis), use of pharmacological intervention with a control (or placebo) group, and that the pharmacological intervention class (e.g. statins) had been used in Phase 2 or three trials in humans for treatment of NAFLD/NASH. Studies were excluded if: not modelling NAFLD/NASH; studies in humans or any animal other than mice and rats; reviews, comments, letters, editorials, meta-analyses, ideas; articles not in English (unless there was an available translation); studies not reporting any relevant outcome metrics (hepatic triglyceride content relative to hepatic protein (e.g. mg/mg or µM/mg), NAFLD Activity Score [Brunt et al., 2011; Kleiner et al., 2005] or any of its components), portal inflammation grade [Brunt et al., 2009], or histological fibrosis stage (0–4); and studies using a pharmacological agent class that had not been used in Phase 2/3 studies in humans for NAFLD.

Abstracts and titles were screened to identify relevant studies using Rayyan (Ouzzani et al., 2016). Potentially relevant studies had their full-text extracted and were assessed against inclusion/exclusion criteria independently by two reviewers, with discrepancies settled by discussion with JPM.

### Data collection

The variables extracted were as follows: phenotypic characteristics of animal model used (sex, diet [including percentage of fat, glucose, fructose, sucrose, and cholesterol in diet], rodent age, genetic alterations, background animal strain); drug treatment (dose, drug class, duration, age at intervention), hepatic triglyceride content and liver histology. Fructose/glucose concentration in diet was collected together as a single data point as they were frequently combined in diets. Liver histology results were extracted where the (human) NAFLD Activity Score (NAS [0–8]) and/or any of its components had been used (steatosis grade [0–3], lobular inflammation [0–3], and ballooning severity [0–2]; portal inflammation severity [0–2]); and/or histological fibrosis stage [0–4]. Studies frequently included multiple cohorts or interventional arms, which were defined as use of a different animal model of NAFLD, a different drug, or a different drug dose. Data were extracted for each cohort or interventional arm separately.

### Quality assessment

Each paper was assessed in the following four areas: use of a protocol, reporting use of randomisation, reporting use of blinding, and a power calculation. ‘Use of a protocol’ assessed the article specifically referring to a protocol that was in place and prior to the start of the study. These were each given a score of 1, and each paper was assigned an overall ‘quality score’. A post-hoc power calculation was performed for each study using the means of each group and a common SD (Cohen, 1988) using the pwr (Champely, 2018) package in R. In addition, a ‘pre-test’ sample size calculation was performed using: the overall effect summary from meta-analysis, power = 80%, and p-value=0.05.

### Shared control group adjustment

Multiple studies used a single placebo (or control) group for several experimental arms. Where possible, the experimental arms were combined into a single experimental cohort and compared to the control group (Higgins and Green, 2011). Where this was not appropriate (e.g. interventions from different drug classes), the control group was divided evenly across interventional groups. Therefore, each control animal was included only once in analyses.

### Data processing

Where possible, drugs were grouped into classes based upon their pharmacological mechanism of action. The majority were well-established classes of drugs: angiotensin receptor blockers, biguanides, dipeptidyl peptidase 4 (DPP4) inhibitors, fibrates, glucagon-like peptide-1 (GLP-1) agonists, statins etc. In some cases there was only a single drug represented in their class, for example: polyphenols – resveratrol, and cholesterol absorption inhibitors – ezetimibe. More novel agents fell into pharmacological classes based on mechanism that are less well established, for example: stearoyl–CoA desaturase-1 inhibitors, or PPARα/δ agonists. Other agents, particularly where the mechanism of action is unclear, were made a class of their own, for example, whilst eicosapentaenoic acid and docosahexaenoic acid are both omega-3 polyunsaturated fatty acids (PUFA), their mechanism is not clear and therefore were classed individually, with other mixtures of PUFA being classed separately. Similarly, berberine and silymarin were classed individually. Where individual bacterial strains were used for probiotics they were classed accordingly (e.g. Lactobacillus sp.), but where a mixture of strains were used a ‘Probiotic (mix)’ category was allocated. For analyses by individual drugs, all agents were separated, though for some drugs (e.g. berberine) this was unchanged from their ‘drug class’ grouping.

Prior to analysis, hepatic triglyceride content was normalized as a percentage of placebo (or control) for each cohort.

Weight, fasting glucose, and fasting insulin of interventional groups were expressed as a proportion difference relative to placebo (e.g. 20% lower fasting glucose in interventional group = 0.8).

All continuous variables were examined for normality using histograms and, where distributions were skewed, variables were logarithmically transformed prior to use in regression analyses.

### Statistical analysis – meta-analysis

Primary outcome was the mean difference in histological fibrosis stage in the interventional group compared to control/placebo. Secondary outcomes were histological features: hepatic triglyceride (TG) content, steatosis grade, lobular inflammation, ballooning, and overall NAS. There was insufficient data to perform meta-analysis for portal inflammation severity.

Random-effects meta-analysis using the Hartung-Knapp-Sidik-Jonkman method was used to calculate mean difference in the outcome measure. Each meta-analysis was run three times, once with subgrouping by drug class, then a sensitivity analysis using subgrouping by drug class after excluding outliers (as described below), and then once using individual drugs. Drug classes, or individual drugs, were only included in meta-analyses where there was data from minimum three unique articles reporting that outcome.

Drugs or drug classes were considered to have a significant effect on the outcome if their 95% CI did not cross zero. Drugs (or drug classes) were also assessed to have greater (or smaller) difference in the outcome measure if their 95% CI did not overlap with the 95% CI of the overall effect estimate. Additionally, for hepatic TG only, drugs were compared to a benchmark of 30% reduction in liver fat. This was based on data from MRI-PDFF in humans that suggests ≥30% reduction in liver fat is associated with a substantial histological response (Jayakumar et al., 2019; Loomba et al., 2020; Stine et al., 2020).

Heterogeneity within drug classes (or individual drugs) and across the whole dataset was reported using Cochran’s Q, Higgin’s and Thompson’s I^2^, and 𝜏^2^. Interpretation of I^2^ was performed according to the Cochrane Handbook where ‘considerable heterogeneity’ refers to P_Q_ <0.05 and I^2^ = 75–100% (Higgins and Green, 2011). Potential outliers were identified using a Baujat plot (Baujat et al., 2002) and by assessment of standard deviation (SD), where all studies with excess contribution to heterogeneity on visual inspection of the Baujat plot or SD >95^th^ centile were excluded in a sensitivity analysis.

Study distribution (‘publication’) bias was assessed using funnel plot with Egger’s test. Given evidence of study distribution bias, Duval and Tweedie’s trim-and-fill procedure (Duval and Tweedie, 2000) was performed to estimate the impact of bias on the overall measure.

### Statistical analysis – meta-regression

Mixed-effects meta-regression was performed to assess which baseline variables were associated with heterogeneity in each outcome measure. Meta-regression was performed using both categorical variables (e.g. drug class, sex, animal background, NAFLD model design) and continuous variables (e.g. percentage of components in diet, age at intervention, drug dose). For each regression analysis, variables were only included where three or more unique articles reported each variable. The number of cohorts included in each regression analysis is reported with their results. Univariable meta-regressions were considered significant where p-value<0.05 and were replicated in more than one outcome metric (e.g. hepatic TG and steatosis grade).

Univariable meta-regression was repeated for weight, glucose, and insulin difference after removal of models causing weight loss. These analyses of weight loss (or gain) with secondary changes in glycaemic control are most relevant to obese or insulin resistant animals. We hypothesised that trends would be strengthened after removal of models that did not recapitulate the metabolic syndrome. Models excluded were: methionine-choline deficient diet (with or without added high-fat), orotic acid, choline deficient diet (with or without added high-fat), and choline deficient L-amino-acid defined diet. Models were excluded irrespective of their genetic background, for example leptin receptor deficiency (*db/db*) plus methionine-choline deficient diet was excluded for this sensitivity analysis. For these three variables, due to replication of testing, statistical significance was set at p-value<0.025.

multiple-variable meta-regression was performed to assess what proportion of between-study heterogeneity could be accounted for by baseline characteristics (using adjusted R^2^). First variables were examined for multicollinearity and where two variables had Pearson correlation >0.6, one was removed. Then, multimodel inference (dmetar::multimodel.inference, RRID:SCR_019054) was used to obtain the model with the best fit for the data. Initially, drug (or drug class) was not included as an input variable as this greatly increased the number of variables and reduced the number of studies for inclusion. The optimum model (defined by the lowest Akaike’s Information Criterion) was then used in multiple-variable meta-regression (known as ‘final model 1’). The robustness of this model was tested using a permutation test (metafor::permutest, RRID:SCR_003450).

This process was repeated to generate ‘final model 2’, by additionally including individual drugs (for TG) or drug class (for steatosis grade and NAS), as input variables in the multimodel inference stage. It was not possible to generate a 2^nd^ multivariable meta-regression model including drug (or drug class) for lobular inflammation, ballooning, and fibrosis due to insufficient data.

For multivariable meta-regression, individual variables were defined as ‘Top predictors’ if they had a predictor importance >0.8 on dmetar::multimodel.inference analysis. Individual variables were considered significant within each model where p-value<0.05. Models were considered to significantly predict outcomes where p-value*<0.05 after use of metafor::permutest.

Statistical analysis was performed using R 4.0.2 for Mac (Harrer et al., 2019; R Core Development team, 2019) with packages dmetar (Harrer et al., 2019), meta (RRID:SCR_019055, [Schwarzer G, 2007]), and metafor (Viechtbauer, 2010). Graphs were also generated using GraphPad Prism (RRID:SCR_002798, v8.0 for Mac, GraphPad Software, La Jolla California, USA).

## Data Availability

The raw dataset used for analysis, including references to individual studies, are available Figure 1-source data 1 and deposited in the Dryad repository at https://doi.org/10.5061/dryad.pzgmsbcgc. R code used for analysis are available in Source code 1. Source data files have been provided for Figures 2-8. The following dataset was generated: MannJP2020Data from: Weight loss, insulin resistance, and study design confound results in a meta-analysis of animal models of fatty liverDryad Digital Repository10.5061/dryad.pzgmsbcgcPMC764739833063664

## Additional files

Source code 1. Code used in analyses. Run in R 4.0.2 with data from Figure 1—source data 1.

Supplementary file 1. Narrative summary of evidence in humans for drug classes included in this meta-analysis. Descriptions of the principle liver-related findings from randomised controlled trials (RCT) both adults and children with NAFLD with references to completed, published studies or protocols for ongoing trials. A dichotomous assessment of whether the drug is associated with weight loss in humans has been added. ACC, Acetyl-CoA carboxylase; ACE, angiotensin-2 converting enzyme; ALT, alanine aminotransferase; ARB, angiotensin receptor blocker; CCR, chemokine receptor; DHA, Docosahexaenoic acid; DPP4, Dipeptidyl peptidase-4; EPA, eicosapentaenoic acid; FXR, Farnesoid X receptor; GLP-1, Glucagon-like peptide-1; LXR, Liver X receptor; MRI, magnetic resonance imaging; NAC, N-acetylcysteine; NAS, NAFLD Activity Score; NASH, non-alcoholic steatohepatitis; PDE, Phosphodiesterase; PDFF, proton-density fat fraction; PPAR, Peroxisome proliferator-activated receptor; PUFA; omega-3 polyunsaturated fatty acid; RAAS, renin-angiotensin-aldosterone system; SCD1, Stearoyl–CoA desaturase-1; SGLT2, Sodium-glucose co-transporter-2; TUDCA, Tauroursodeoxycholic acid; and UDCA, Ursodeoxycholic acid.

Supplementary file 2. Systematic review protocol. Prospectively registered on SyRF in August 2017.

Transparent reporting form

## References

[bib1] Angulo P, Kleiner DE, Dam-Larsen S, Adams LA, Bjornsson ES, Charatcharoenwitthaya P, Mills PR, Keach JC, Lafferty HD, Stahler A, Haflidadottir S, Bendtsen F (2015). Liver fibrosis, but no other histologic features, is associated with Long-term outcomes of patients with nonalcoholic fatty liver disease. Gastroenterology.

[bib2] Anstee QM, Goldin RD (2006). Mouse models in non-alcoholic fatty liver disease and steatohepatitis research. International Journal of Experimental Pathology.

[bib3] Armstrong MJ, Gaunt P, Aithal GP, Barton D, Hull D, Parker R, Hazlehurst JM, Guo K, Abouda G, Aldersley MA, Stocken D, Gough SC, Tomlinson JW, Brown RM, Hübscher SG, Newsome PN (2015). Liraglutide safety and efficacy in patients with non-alcoholic steatohepatitis (LEAN): a multicentre, double-blind, randomised, placebo-controlled phase 2 study. The Lancet.

[bib4] Bahor Z, Liao J, Macleod MR, Bannach-Brown A, McCann SK, Wever KE, Thomas J, Ottavi T, Howells DW, Rice A, Ananiadou S, Sena E (2017). Risk of Bias reporting in the recent animal focal cerebral ischaemia literature. Clinical Science.

[bib5] Baker M (2016). 1,500 scientists lift the lid on reproducibility. Nature.

[bib6] Balmer ML, Slack E, de Gottardi A, Lawson MA, Hapfelmeier S, Miele L, Grieco A, Van Vlierberghe H, Fahrner R, Patuto N, Bernsmeier C, Ronchi F, Wyss M, Stroka D, Dickgreber N, Heim MH, McCoy KD, Macpherson AJ (2014). The liver may act as a firewall mediating mutualism between the host and its gut commensal Microbiota. Science Translational Medicine.

[bib7] Barchetta I, Del Ben M, Angelico F, Di Martino M, Fraioli A, La Torre G, Saulle R, Perri L, Morini S, Tiberti C, Bertoccini L, Cimini FA, Panimolle F, Catalano C, Baroni MG, Cavallo MG (2016). No effects of oral vitamin D supplementation on non-alcoholic fatty liver disease in patients with type 2 diabetes: a randomized, double-blind, placebo-controlled trial. BMC Medicine.

[bib8] Baujat B, Mahé C, Pignon JP, Hill C (2002). A graphical method for exploring heterogeneity in meta-analyses: application to a meta-analysis of 65 trials. Statistics in Medicine.

[bib9] Brenner DA (2018). Of Mice and Men and Nonalcoholic Steatohepatitis. Hepatology.

[bib10] Brunt EM, Kleiner DE, Wilson LA, Unalp A, Behling CE, Lavine JE, Neuschwander-Tetri BA, NASH Clinical Research NetworkA list of members of the Nonalcoholic Steatohepatitis Clinical Research Network can be found in the Appendix (2009). Portal chronic inflammation in nonalcoholic fatty liver disease (NAFLD): a histologic marker of advanced NAFLD-Clinicopathologic correlations from the nonalcoholic steatohepatitis clinical research network. Hepatology.

[bib11] Brunt EM, Kleiner DE, Wilson LA, Belt P, Neuschwander-Tetri BA, NASH Clinical Research Network (CRN) (2011). Nonalcoholic fatty liver disease (NAFLD) activity score and the histopathologic diagnosis in NAFLD: distinct clinicopathologic meanings. Hepatology.

[bib12] Budas G, Karnik S, Jonnson T, Shafizadeh T, Watkins S, Breckenridge D, Tumas D (2016). Reduction of liver steatosis and fibrosis with an Ask1 inhibitor in a murine model of nash is accompanied by improvements in cholesterol, bile acid and lipid metabolism. Journal of Hepatology.

[bib13] Byrne CD, Targher G (2015). NAFLD: a multisystem disease. Journal of Hepatology.

[bib14] Chalasani N, Younossi Z, Lavine JE, Charlton M, Cusi K, Rinella M, Harrison SA, Brunt EM, Sanyal AJ (2018). The diagnosis and management of nonalcoholic fatty liver disease: practice guidance from the american association for the study of liver diseases. Hepatology.

[bib15] Champely S (2018). R Package.

[bib16] Chella Krishnan K, Kurt Z, Barrere-Cain R, Sabir S, Das A, Floyd R, Vergnes L, Zhao Y, Che N, Charugundla S, Qi H, Zhou Z, Meng Y, Pan C, Seldin MM, Norheim F, Hui S, Reue K, Lusis AJ, Yang X (2018). Integration of Multi-omics data from mouse diversity panel highlights mitochondrial dysfunction in Non-alcoholic fatty liver disease. Cell Systems.

[bib17] Cohen J (1988). Statistical Power Analysis for the Behavioral Sciences.

[bib18] Cusi K, Orsak B, Bril F, Lomonaco R, Hecht J, Ortiz-Lopez C, Tio F, Hardies J, Darland C, Musi N, Webb A, Portillo-Sanchez P (2016). Long-Term pioglitazone treatment for patients with nonalcoholic steatohepatitis and prediabetes or type 2 diabetes mellitus: a randomized trial. Annals of Internal Medicine.

[bib19] Dabbaghmanesh MH, Danafar F, Eshraghian A, Omrani GR (2018). Vitamin D supplementation for the treatment of non-alcoholic fatty liver disease: a randomized double blind placebo controlled trial. Diabetes & Metabolic Syndrome: Clinical Research & Reviews.

[bib20] de Vries RB, Hooijmans CR, Tillema A, Leenaars M, Ritskes-Hoitinga M (2014). Updated version of the embase search filter for animal studies. Laboratory Animals.

[bib21] Denayer T, Stöhr T, Van Roy M (2014). Animal models in translational medicine: validation and prediction. New Horizons in Translational Medicine.

[bib22] Duval S, Tweedie R (2000). Trim and fill: a simple funnel-plot-based method of testing and adjusting for publication Bias in meta-analysis. Biometrics.

[bib23] Ekstedt M, Hagström H, Nasr P, Fredrikson M, Stål P, Kechagias S, Hultcrantz R (2015). Fibrosis stage is the strongest predictor for disease-specific mortality in NAFLD after up to 33 years of follow-up. Hepatology.

[bib24] Fabbrini E, Mohammed BS, Korenblat KM, Magkos F, McCrea J, Patterson BW, Klein S (2010). Effect of fenofibrate and niacin on intrahepatic triglyceride content, very low-density lipoprotein kinetics, and insulin action in obese subjects with nonalcoholic fatty liver disease. The Journal of Clinical Endocrinology & Metabolism.

[bib25] Farrell G, Schattenberg JM, Leclercq I, Yeh MM, Goldin R, Teoh N, Schuppan D (2019). Mouse models of nonalcoholic steatohepatitis: toward optimization of their relevance to human nonalcoholic steatohepatitis. Hepatology.

[bib26] Flórez-Vargas O, Brass A, Karystianis G, Bramhall M, Stevens R, Cruickshank S, Nenadic G (2016). Bias in the reporting of sex and age in biomedical research on mouse models. eLife.

[bib27] Friedman SL, Neuschwander-Tetri BA, Rinella M, Sanyal AJ (2018). Mechanisms of NAFLD development and therapeutic strategies. Nature Medicine.

[bib28] Hackam DG, Redelmeier DA (2006). Translation of research evidence from animals to humans. Jama.

[bib29] Harrer M, Cuijpers P, Furukawa TA, Ebert DD (2019). Doing Meta-Analysis in R: A Hands-on Guide.

[bib30] Harrison SA, Abdelmalek MF, Caldwell S, Shiffman ML, Diehl AM, Ghalib R, Lawitz EJ, Rockey DC, Schall RA, Jia C, McColgan BJ, McHutchison JG, Subramanian GM, Myers RP, Younossi Z, Ratziu V, Muir AJ, Afdhal NH, Goodman Z, Bosch J, Sanyal AJ, Us G, Investigators G-U- (2018). Simtuzumab Is Ineffective for Patients With Bridging Fibrosis or Compensated Cirrhosis Caused by Nonalcoholic Steatohepatitis. Gastroenterology.

[bib31] Henderson VC, Demko N, Hakala A, MacKinnon N, Federico CA, Fergusson D, Kimmelman J (2015). A meta-analysis of threats to valid clinical inference in preclinical research of sunitinib. eLife.

[bib32] Higgins JPT, Green S (2011). Cochrane Handbook for Systematic Reviews of Interventions.

[bib33] Homanics GE, Quinlan JJ, Firestone LL (1999). Pharmacologic and behavioral responses of inbred C57BL/6J and strain 129/SvJ mouse lines. Pharmacology Biochemistry and Behavior.

[bib34] Hooijmans CR, Tillema A, Leenaars M, Ritskes-Hoitinga M (2010). Enhancing search efficiency by means of a search filter for finding all studies on animal experimentation in PubMed. Laboratory Animals.

[bib35] Howells DW, Sena ES, Macleod MR (2014). Bringing rigour to translational medicine. Nature Reviews Neurology.

[bib36] Hui ST, Parks BW, Org E, Norheim F, Che N, Pan C, Castellani LW, Charugundla S, Dirks DL, Psychogios N, Neuhaus I, Gerszten RE, Kirchgessner T, Gargalovic PS, Lusis AJ (2015). The genetic architecture of NAFLD among inbred strains of mice. eLife.

[bib37] Hui ST, Kurt Z, Tuominen I, Norheim F, C.Davis R, Pan C, Dirks DL, Magyar CE, French SW, Chella Krishnan K, Sabir S, Campos-Pérez F, Méndez-Sánchez N, Macías-Kauffer L, León-Mimila P, Canizales-Quinteros S, Yang X, Beaven SW, Huertas-Vazquez A, Lusis AJ (2018). The Genetic Architecture of Diet-Induced Hepatic Fibrosis in Mice. Hepatology.

[bib38] Jayakumar S, Middleton MS, Lawitz EJ, Mantry PS, Caldwell SH, Arnold H, Mae Diehl A, Ghalib R, Elkhashab M, Abdelmalek MF, Kowdley KV, Stephen Djedjos C, Xu R, Han L, Mani Subramanian G, Myers RP, Goodman ZD, Afdhal NH, Charlton MR, Sirlin CB, Loomba R (2019). Longitudinal correlations between MRE, MRI-PDFF, and liver histology in patients with non-alcoholic steatohepatitis: analysis of data from a phase II trial of selonsertib. Journal of Hepatology.

[bib39] Kaden-Volynets V, Basic M, Neumann U, Pretz D, Rings A, Bleich A, Bischoff SC (2019). Lack of liver steatosis in germ-free mice following hypercaloric diets. European Journal of Nutrition.

[bib40] Keech A, Simes RJ, Barter P, Best J, Scott R, Taskinen MR, Forder P, Pillai A, Davis T, Glasziou P, Drury P, Kesäniemi YA, Sullivan D, Hunt D, Colman P, d'Emden M, Whiting M, Ehnholm C, Laakso M, FIELD study investigators (2005). Effects of long-term fenofibrate therapy on cardiovascular events in 9795 people with type 2 diabetes mellitus (the FIELD study): randomised controlled trial. Lancet.

[bib41] Kleiner DE, Brunt EM, Van Natta M, Behling C, Contos MJ, Cummings OW, Ferrell LD, Liu Y-C, Torbenson MS, Unalp-Arida A, Yeh M, McCullough AJ, Sanyal AJ, Nonalcoholic Steatohepatitis Clinical Research Network (2005). Design and validation of a histological scoring system for nonalcoholic fatty liver disease. Hepatology.

[bib42] Koutoukidis DA, Astbury NM, Tudor KE, Morris E, Henry JA, Noreik M, Jebb SA, Aveyard P (2019). Association of weight loss interventions with changes in biomarkers of nonalcoholic fatty liver disease: a systematic review and Meta-analysis. JAMA Internal Medicine.

[bib43] Landis SC, Amara SG, Asadullah K, Austin CP, Blumenstein R, Bradley EW, Crystal RG, Darnell RB, Ferrante RJ, Fillit H, Finkelstein R, Fisher M, Gendelman HE, Golub RM, Goudreau JL, Gross RA, Gubitz AK, Hesterlee SE, Howells DW, Huguenard J, Kelner K, Koroshetz W, Krainc D, Lazic SE, Levine MS, Macleod MR, McCall JM, Moxley RT, Narasimhan K, Noble LJ, Perrin S, Porter JD, Steward O, Unger E, Utz U, Silberberg SD (2012). A call for transparent reporting to optimize the predictive value of preclinical research. Nature.

[bib44] Lassailly G, Caiazzo R, Buob D, Pigeyre M, Verkindt H, Labreuche J, Raverdy V, Leteurtre E, Dharancy S, Louvet A, Romon M, Duhamel A, Pattou F, Mathurin P (2015). Bariatric surgery reduces features of nonalcoholic steatohepatitis in morbidly obese patients. Gastroenterology.

[bib45] Lee Y, Doumouras AG, Yu J, Brar K, Banfield L, Gmora S, Anvari M, Hong D (2019). Complete resolution of nonalcoholic fatty liver disease after bariatric surgery: a systematic review and Meta-analysis. Clinical Gastroenterology and Hepatology.

[bib46] Liu X, Gershenfeld HK (2001). Genetic differences in the tail-suspension test and its relationship to imipramine response among 11 inbred strains of mice. Biological Psychiatry.

[bib47] Loomba R, Neuschwander‐Tetri BA, Sanyal A, Chalasani N, Diehl AM, Terrault N, Kowdley K, Dasarathy S, Kleiner D, Behling C, Lavine J, Van Natta M, Middleton M, Tonascia J, Sirlin C, Allende D, Dasarathy S, McCullough AJ, Penumatsa R, Dasarathy J, Lavine JE, Abdelmalek MF, Bashir M, Buie S, Diehl AM, Guy C, Kigongo C, Kopping M, Malik D, Piercy D, Chalasani N, Cummings OW, Gawrieh S, Ragozzino L, Sandrasegaran K, Vuppalanchi R, Brunt EM, Cattoor T, Carpenter D, Freebersyser J, King D, Lai J, Neuschwander‐Tetri BA, Siegner J, Stewart S, Torretta S, Wriston K, Gonzalez MC, Davila J, Jhaveri M, Kowdley KV, Mukhtar N, Ness E, Poitevin M, Quist B, Soo S, Ang B, Behling C, Bhatt A, Loomba R, Middleton MS, Sirlin C, Akhter MF, Bass NM, Brandman D, Gill R, Hameed B, Maher J, Terrault N, Ungermann A, Yeh M, Boyett S, Contos MJ, Kirwin S, Luketic VAC, Puri P, Sanyal AJ, Schlosser J, Siddiqui MS, Yost‐Schomer L, Brunt EM, Fowler K, Kleiner DE, Doo EC, Hall S, Hoofnagle JH, Robuck PR, Sherker AH, Torrance R, Belt P, Clark JM, Dodge J, Donithan M, Isaacson M, Lazo M, Meinert J, Miriel L, Sharkey EP, Smith J, Smith M, Sternberg A, Tonascia J, Van Natta ML, Wagoner A, Wilson LA, Yamada G, Yates K, Covarrubias Y, Gamst A, Hamilton G, Henderson W, Hooker J, Lavine JE, Loomba R, Middleton MS, Schlein A, Schwimmer JB, Shen W, Sirlin C, Wolfson T (2020). Multicenter validation of association between decline in mri‐pdff and histologic response in NASH. Hepatology.

[bib48] Macleod MR, O'Collins T, Horky LL, Howells DW, Donnan GA (2005). Systematic review and metaanalysis of the efficacy of FK506 in experimental stroke. Journal of Cerebral Blood Flow & Metabolism.

[bib49] Macleod MR, Lawson McLean A, Kyriakopoulou A, Serghiou S, de Wilde A, Sherratt N, Hirst T, Hemblade R, Bahor Z, Nunes-Fonseca C, Potluru A, Thomson A, Baginskaite J, Baginskitae J, Egan K, Vesterinen H, Currie GL, Churilov L, Howells DW, Sena ES (2015). Risk of Bias in reports of in vivo research: a focus for improvement. PLOS Biology.

[bib50] Mann JP, De Vito R, Mosca A, Alisi A, Armstrong MJ, Raponi M, Baumann U, Nobili V (2016). Portal inflammation is independently associated with fibrosis and metabolic syndrome in pediatric nonalcoholic fatty liver disease. Hepatology.

[bib51] Martin MD, Danahy DB, Hartwig SM, Harty JT, Badovinac VP (2017). Revealing the complexity in CD8 T cell responses to infection in inbred C57B/6 versus outbred swiss mice. Frontiers in Immunology.

[bib52] Mazagova M, Wang L, Anfora AT, Wissmueller M, Lesley SA, Miyamoto Y, Eckmann L, Dhungana S, Pathmasiri W, Sumner S, Westwater C, Brenner DA, Scmiabl B (2015). Commensal Microbiota is hepatoprotective and prevents liver fibrosis in mice. The FASEB Journal.

[bib53] Mestas J, Hughes CC (2004). Of mice and not men: differences between mouse and human immunology. The Journal of Immunology.

[bib54] Oscarsson J, Önnerhag K, Risérus U, Sundén M, Johansson L, Jansson PA, Moris L, Nilsson PM, Eriksson JW, Lind L (2018). Effects of free omega-3 carboxylic acids and fenofibrate on liver fat content in patients with hypertriglyceridemia and non-alcoholic fatty liver disease: a double-blind, randomized, placebo-controlled study. Journal of Clinical Lipidology.

[bib55] Ouzzani M, Hammady H, Fedorowicz Z, Elmagarmid A (2016). Rayyan—a web and mobile app for systematic reviews. Systematic Reviews.

[bib56] Perel P, Roberts I, Sena E, Wheble P, Briscoe C, Sandercock P, Macleod M, Mignini LE, Jayaram P, Khan KS (2007). Comparison of treatment effects between animal experiments and clinical trials: systematic review. BMJ.

[bib57] Prescott MJ, Lidster K (2017). Improving quality of science through better animal welfare: the NC3Rs strategy. Lab Animal.

[bib58] R Core Development team (2019).

[bib59] Rakha EA, Adamson L, Bell E, Neal K, Ryder SD, Kaye PV, Aithal GP (2010). Portal inflammation is associated with advanced histological changes in alcoholic and non-alcoholic fatty liver disease. Journal of Clinical Pathology.

[bib60] Rangarajan A, Weinberg RA (2003). Comparative biology of mouse versus human cells: modelling human Cancer in mice. Nature Reviews Cancer.

[bib61] Ratziu V, Sanyal AJ, Loomba R, Rinella M, Harrison S, Anstee QM, Goodman Z, Bedossa P, MacConell L, Shringarpure R, Shah A, Younossi Z (2019). REGENERATE: design of a pivotal, randomised, phase 3 study evaluating the safety and efficacy of obeticholic acid in patients with fibrosis due to nonalcoholic steatohepatitis. Contemporary Clinical Trials.

[bib62] Sanyal AJ, Chalasani N, Kowdley KV, McCullough A, Diehl AM, Bass NM, Neuschwander-Tetri BA, Lavine JE, Tonascia J, Unalp A, Van Natta M, Clark J, Brunt EM, Kleiner DE, Hoofnagle JH, Robuck PR, NASH CRN (2010). Pioglitazone, vitamin E, or placebo for nonalcoholic steatohepatitis. New England Journal of Medicine.

[bib63] Sanyal AJ, Abdelmalek MF, Suzuki A, Cummings OW, Chojkier M, EPE-A Study Group (2014). No significant effects of ethyl-eicosapentanoic acid on histologic features of nonalcoholic steatohepatitis in a phase 2 trial. Gastroenterology.

[bib64] Sanyal AJ (2019). Past, present and future perspectives in nonalcoholic fatty liver disease. Nature Reviews Gastroenterology & Hepatology.

[bib65] Schwarzer G O (2007). R News.

[bib66] Sena ES, van der Worp HB, Bath PM, Howells DW, Macleod MR (2010). Publication Bias in reports of animal stroke studies leads to major overstatement of efficacy. PLOS Biology.

[bib67] Harrison SA, Wong VW, Okanoue T, Bzowej N, Vuppalanchi R, Younes Z, Kohli A, Sarin S, Caldwell SH, Alkhouri N, Shiffman ML, Camargo M, Li G, Kersey K, Jia C, Zhu Y, Djedjos CS, Subramanian GM, Myers RP, Gunn N, Sheikh A, Anstee QM, Romero-Gomez M, Trauner M, Goodman Z, Lawitz EJ, Younossi Z, STELLAR-3 and STELLAR-4 Investigators (2020). Selonsertib for patients with bridging fibrosis or compensated cirrhosis due to NASH: results from randomized phase III STELLAR trials. Journal of Hepatology.

[bib68] Stine JG, Munaganuru N, Barnard A, Wang JL, Kaulback K, Argo CK, Singh S, Fowler KJ, Sirlin CB, Loomba R (2020). Change in MRI-PDFF and histologic response in patients with nonalcoholic steatohepatitis: a systematic review and Meta-Analysis. Clinical Gastroenterology and Hepatology.

[bib69] Tsilidis KK, Panagiotou OA, Sena ES, Aretouli E, Evangelou E, Howells DW, Al-Shahi Salman R, Macleod MR, Ioannidis JP (2013). Evaluation of excess significance Bias in animal studies of neurological diseases. PLOS Biology.

[bib70] van der Worp HB, Howells DW, Sena ES, Porritt MJ, Rewell S, O'Collins V, Macleod MR (2010). Can animal models of disease reliably inform human studies?. PLOS Medicine.

[bib71] Viechtbauer W (2010). Conducting Meta-Analyses in R with the metafor package. Journal of Statistical Software.

[bib72] von Herrath M, Pagni PP, Grove K, Christoffersson G, Tang-Christensen M, Karlsen AE, Petersen JS (2019). Case reports of Pre-clinical replication studies in metabolism and diabetes. Cell Metabolism.

[bib73] Watzlawick R, Antonic A, Sena ES, Kopp MA, Rind J, Dirnagl U, Macleod M, Howells DW, Schwab JM (2019). Outcome heterogeneity and Bias in acute experimental spinal cord injury: a meta-analysis. Neurology.

[bib74] Younossi Z, Anstee QM, Marietti M, Hardy T, Henry L, Eslam M, George J, Bugianesi E (2018). Global burden of NAFLD and NASH: trends, predictions, risk factors and prevention. Nature Reviews Gastroenterology & Hepatology.

[bib75] Younossi ZM, Golabi P, de Avila L, Paik JM, Srishord M, Fukui N, Qiu Y, Burns L, Afendy A, Nader F (2019). The global epidemiology of NAFLD and NASH in patients with type 2 diabetes: A systematic review and meta-analysis. Journal of Hepatology.

