# Supplementary material for: Weight loss, insulin resistance, and study design confound results in a meta-analysis of animal models of fatty liver
Source: eLife. 2020 Oct 16;9:e56573. doi: 10.7554/eLife.56573 (PMC7647398; doi:10.7554/eLife.56573)
Supplement: Supplementary file 1. — Descriptions of the principle liver-related findings from randomised controlled trials (RCT) both adults and children with NAFLD with references to completed, published studies or protocols for ongoing trials. A dichotomous assessment of whether the drug is associated with weight loss in humans has been added. ACC, Acetyl-CoA carboxylase; ACE, angiotensin-2 converting enzyme; ALT, alanine aminotransferase; ARB, angiotensin receptor blocker; CCR, chemokine receptor; DHA, Docosahexaenoic acid; DPP4, Dipeptidyl peptidase-4; EPA, eicosapentaenoic acid; FXR, Farnesoid X receptor; GLP-1, Glucagon-like peptide-1; LXR, Liver X receptor; MRI, magnetic resonance imaging; NAC, N-acetylcysteine; NAS, NAFLD Activity Score; NASH, non-alcoholic steatohepatitis; PDE, Phosphodiesterase; PDFF, proton-density fat fraction; PPAR, Peroxisome proliferator-activated receptor; PUFA; omega-3 polyunsaturated fatty acid; RAAS, renin-angiotensin-aldosterone system; SCD1, Stearoyl–CoA desaturase-1; SGLT2, Sodium-glucose co-transporter-2; TUDCA, Tauroursodeoxycholic acid; and UDCA, Ursodeoxycholic acid. [file elife-56573-supp1.docx]

|  | **Description & mechanism** | **Efficacy in on NAFLD in humans** | **Weight loss in humans** | **References** |
| --- | --- | --- | --- | --- |
| **ACC inhibitor** | Acetyl-CoA carboxylase (ACC) is a cytosolic enzyme that is the rate limiting step in *de novo* synthesis of fatty acids. Inhibition aims to reduce hepatic lipid by reducing hepatic *de novo* lipogenesis. | Phase 2: reduction of liver fat but increase in plasma triglycerides | No | [(Alkhouri et al., 2020)](https://paperpile.com/c/Q7lYca/qsjA) |
| **ACE inhibitor & Angiotensin Receptor Blockers (ARB)** | Angiotensin-converting enzyme (ACE) 2 is a critical regulator of the renin-angiotensin aldosterone system (RAAS), with the angiotensin receptor being the principal downstream target of the pathway. The RAAS had been implicated in NASH and fibrosis. | Phase 2: insufficient recruitment to determine effect due to many patients already on ACEi or ARB  Phase 2: improvement in ALT & fibrosis on valsartan | No | [(McPherson et al., 2017)](https://paperpile.com/c/Q7lYca/SSxV) |
| **Alpha glucosidase inhibitor** | Acarbose is an inhibitor of intestinal alpha-glucosidase, which releases glucose from starch and disaccharides. Its use is associated with improved glycaemic control though generally poorly tolerated due to gastrointestinal side-effects. | Phase 2: improvement in biochemical indices of NAFLD. No histological data.    Trial in paediatric NAFLD failed to recruit. | No | [(Hajiaghamohammadi et al., 2013)](https://paperpile.com/c/Q7lYca/QJJ6)    https://clinicaltrials.gov/ct2/show/NCT00677521 |
| **Berberine** | Berberine is an organic compound found in many plants that has been traditionally used to treat complications of the metabolic syndrome. | Phase 2: improvement in hepatic fat content on magnetic resonance spectroscopy | No | [(Yan et al., 2015)](https://paperpile.com/c/Q7lYca/0lCh) |
| **Bifidobacterium sp.** | *Bifidobacterium* belongs to the *Bifidobacteria* genera and is a frequently used probiotic either by itself or in combination with other strains. | Trial in progress | No | [(Scorletti et al., 2018)](https://paperpile.com/c/Q7lYca/B5UE) |
| **Biguanide** | Metformin is an antihyperglycaemic agent which improves glucose tolerance and reduces body weight in patients with type 2 diabetes. It decreases hepatic glucose production and intestinal absorption of glucose, and improves insulin sensitivity by increasing peripheral glucose uptake and utilization. | Phase 2: improvement in histological NAS and liver transaminases  Phase 3: no significant improvement in histological features of NAFLD or liver transaminases | Yes | [(Haukeland et al., 2009; Loomba et al., 2009)](https://paperpile.com/c/Q7lYca/dsRg+iPW1) |
| **Caspase inhibitor** | Caspases, key mediators of apoptosis, are a family of proteases that cleave their substrates either to cause cell death or to activate cytokines as part of an immune response. Emricasan is an irreversible caspase inhibitor. | Phase 2: no improvement in liver histology in patients with NASH fibrosis on emricasan  Phase 2: no improvement in hepatic venous pressure gradient or clinical outcomes in patients with NASH-related cirrhosis and severe portal hypertension  Phase 2: improvement in liver transaminases and biomarkers | No | [(Garcia-Tsao et al., 2020; Harrison et al., 2020; Shiffman et al., 2019)](https://paperpile.com/c/Q7lYca/OzKz+TmW9+Ovtf) |
| **CCR2/CCR5 antagonist** | Cenicriviroc is a dual antagonist of C-C motif chemokine receptor (CCR) types 2 and 5. Blockade has been shown to have anti-inflammatory and antifibrotic properties. | Phase 2: improvement in fibrosis and no significant change in NAS on cenicriviroc  Phase 3 in progress | No | [(Friedman et al., 2018)](https://paperpile.com/c/Q7lYca/w5tl)  https://clinicaltrials.gov/ct2/show/NCT03028740 |
| **Cholesterol Absorption Inhibitor** | Ezetimibe is a lipid-lowering compound that inhibits intestinal absorption of cholesterol by binding to Niemann–Pick C1-like 1, a cholesterol transporter, and therefore reduces the delivery of cholesterol to the liver. | Phase 2: improvement in biochemical indices of NAFLD on ezetimibe. No histological data.    Phase 2: no significant reduction in hepatic fat assessed by MRI proton density fat fraction (PDFF) and liver histology on ezetimibe | No | [(Hajiaghamohammadi et al., 2013; Loomba et al., 2015)](https://paperpile.com/c/Q7lYca/bEHQ+QJJ6) |
| **Curcumin** | Curcumin is the active ingredient in turmeric and is widely used as a traditional herbal remedy for a variety of conditions. | Phase 2: improvement in liver fat assessed ultrasonographically and biochemical markers of NAFLD  Phase 2: improvement in liver fat assessed ultrasonographically and liver transaminases  Further trials in progress | No | [(Panahi et al., 2017; Rahmani et al., 2016)](https://paperpile.com/c/Q7lYca/m6nt+DHwC)    <https://clinicaltrials.gov/ct2/show/NCT04109742>  https://clinicaltrials.gov/ct2/show/NCT03864783 |
| **Docosahexaenoic acid** | Docosahexaenoic acid (DHA) is an omega-3 fatty acid which reduces plasma triglycerides. | Phase 2: improvement in liver steatosis assessed ultrasonographically and insulin sensitivity (in children). No change in liver transaminases.  Phase 2 trial in progress | No | [(Nobili et al., 2011)](https://paperpile.com/c/Q7lYca/Vyxp)    https://clinicaltrials.gov/ct2/show/NCT04198805 |
| **DPP4 inhibitor** | DPP-4 inhibition increases incretin levels through reduced degradation of circulating GLP-1. This results in increased insulin secretion, decreases gastric emptying, and decreases blood glucose levels. | Phase 2: sitagliptin was not significantly better than placebo in reducing hepatic fat or transaminases  Phase 4: improvement in hepatic fat content and liver transaminases on vildagliptin | Yes | [(Cui et al., 2016; Macauley et al., 2015)](https://paperpile.com/c/Q7lYca/yNL1+28mR) |
| **Eicosapentaenoic acid** | Eicosapentaenoic acid (EPA) is an omega-3 polyunsaturated fatty acid that reduces hypertriglyceridemia. | Phase 2: no improvement in histological features of NASH or liver transaminases | No | [(Sanyal et al., 2014)](https://paperpile.com/c/Q7lYca/FCZH)      https://pubmed.ncbi.nlm.nih.gov/24818764/ |
| **Fibrates** | Fibrates activate peroxisome proliferator-activated receptor α (PPARα) to alter lipid metabolism and treat primary hypercholesterolemia, mixed dyslipidemia, and severe hypertriglyceridemia. | Phase 2: no improvement in liver fat assessed by MRI-PDFF on fenofibrate  Phase 2: improvement in liver trasnaminsases on fenofibrate  Phase 2: no improvement in intrahepatic triglyceride content on fenofibrate | No | [(El-Haggar and Mostafa, 2015; Fabbrini et al., 2010; Oscarsson et al., 2018; Yaghoubi et al., 2017)](https://paperpile.com/c/Q7lYca/kQ7P+ecZv+whAt+5zpz) |
| **FXR agonist** | FXR agonists promote insulin sensitivity and decrease hepatic gluconeogenesis and circulating triglycerides. Obeticholic acid, a synthetic variant of the natural bile acid chenodeoxycholic acid, is a potent FXR activator. | Phase 2: improvement in NAS on obeticholic acid  Phase 2: reduction in markers of liver inflammation and fibrosis on obeticholic acid  Phase 3 trials in progress | No | [(Mudaliar et al., 2013; Neuschwander-Tetri et al., 2015)](https://paperpile.com/c/Q7lYca/RlX0+1bX7)  <https://clinicaltrials.gov/ct2/show/study/NCT03439254>  https://clinicaltrials.gov/ct2/show/NCT02548351 |
| **GLP-1 agonist** | GLP-1 agonists mimic the effects of endogenous GLP-1, thus enhancing glucose-stimulated insulin secretion and lower blood glucose levels. | Phase 2: improvement of histological steatohepatitis on liraglutide  Phase 4: no reduction in hepatic fat on magnetic resonance spectroscopy on liraglutide | Yes | [(Armstrong et al., 2015; Bizino et al., 2020)](https://paperpile.com/c/Q7lYca/jAo5+Tklt) |
| **Lactobacillus sp.** | *Lactobacillus* is a genus of Gram‐positive bacteria which convert sugars into lactic acid. It is one of the commonly used probiotics. | Pilot studies: improvement in liver transaminases on lactobacillus rhamnosus  Phase 2 trial in progress | No | [(Abdel Monem, 2017; Tenorio-Jiménez et al., 2018; Vajro et al., 2011)](https://paperpile.com/c/Q7lYca/YHah+lOG0+pyg1) |
| **LXR inhibition** | LXR inhibition reduces the synthesis of fatty acid but expedites lipid oxidation by inhibiting the LXR-a activity and decreasing the expression of SREBP-1c within the liver. | Phase 2: improvement in hepatic fat content on magnetic resonance spectroscopy on oltipraz  Phase 3 trial in progress | No | [(Kim et al., 2017)](https://paperpile.com/c/Q7lYca/Qu9m)  https://clinicaltrials.gov/ct2/show/NCT04142749 |
| **N-acetylcysteine** | N-acetylcysteine (NAC) is a glutathione precursor which increases glutathione levels in hepatocytes. Increased glutathione levels limit the production of reactive oxygen species which cause hepatocellular injury. | Pilot study: No significant change in steatosis assessed ultrasonographically. Improvement in liver transaminases  Phase 2 trial in progress (in children) | No | [(Khoshbaten et al., 2010)](https://paperpile.com/c/Q7lYca/GzUI)  https://clinicaltrials.gov/ct2/show/NCT02117700 |
| **Omega-3 polyunsaturated fatty acids (mix)** | n-3 polyunsaturated fatty acids are thought to be able to affect insulin resistance, lipogenesis, and inflammation, which are features of nonalcoholic steatohepatitis. | Phase 3: PUFA supplementation given, plasma increase in PUFA seen in both treatment and placebo group suggesting off-protocol intake. Plasma increase of PUFAs was correlated with NAS improvement.  Phase 2: no improvement in NAS or biochemical markers on PUFA. Improvement in hepatic fat assessed by MRI and computer assisted fat morphometry.  Phase 4: no evidence of improvement in liver fat on DHA+EPA. Showed a trend toward a decrease in liver fat percentage with DHA+EPA treatment, but there was strong evidence for contamination with DHA and EPA enrichment in the placebo group and poor adherence to DHA+EPA intervention in the treatment arm. | No | [(Argo et al., 2015; Nogueira et al., 2016; Scorletti et al., 2014)](https://paperpile.com/c/Q7lYca/TvhF+zacI+tGrc) |
| **PDE inhibitor (Pentoxifylline)** | Pentoxifylline is a methylxanthine derivative that increases red blood cell flexibility, reduces blood viscosity, and decreases platelet aggregation. Therapy with pentoxifylline has been associated with a significant reduction of oxidized fatty acids. | Phase 2: no significant differences in improvement in liver transaminases and histological features of NASH when compared to placebo  Phase 2: improvement in overall NAS, steatosis, lobular inflammation and fibrosis assessed histologically. No improvement in hepatocellular ballooning. | No | [(Van Wagner et al., 2011; Zein et al., 2011)](https://paperpile.com/c/Q7lYca/mQ8m+gTiF) |
| **Polyphenol (Resveratrol)** | Resveratrol is a phytoalexin found in many plants including grapes, peanuts and berries. Resveratrol activates SIRT1 and therefore is thought to benefit diseases affected by abnormal metabolic control, inflammation, and cell cycle defects. | Phase 2: no improvement in hepatic steatosis or biochemical parameters of NAFLD  Phase 2: no improvement in hepatic fat content assessed by magnetic resonance spectroscopy  Phase 2: no improvement in hepatic fat content assessed by magnetic resonance spectroscopy. Significant increase in liver transaminases  Phase 2: improvement in biochemical parameters and hepatic steatosis grade | No | [(Asghari et al., 2018; Chachay et al., 2014; Faghihzadeh et al., 2014; Kantartzis et al., 2018)](https://paperpile.com/c/Q7lYca/xmEs+51V1+jrb3+ZF39) |
| **PPARalpha-delta agonist** | Elafibranor is a dual PPARalpha-delta agonist which causes a reduction in hepatic expression of pro-inflammatory genes and genes involved in fibrogenesis. | Phase 2: improvement in NASH on elafibranor  Phase 2 trial in progress  Phase 3 trial in progress | No | [(Ratziu et al., 2016)](https://paperpile.com/c/Q7lYca/bPuh)  https://clinicaltrials.gov/ct2/show/NCT03883607  https://clinicaltrials.gov/ct2/show/NCT02704403 |
| **Probiotic (mix)** | Probiotics are living, non-pathogenic microorganisms. Many of the probiotics used clinically are part of normal human gut flora. They are used medically and commercially for mainly gastrointestinal illnesses. The exact mechanism of action of probiotics is unknown but hypotheses include preventing growth of pathogenic bacteria and anti-inflammatory effects. | Phase 2: improvement in fatty liver severity assessed ultrasonographically on VSL#3. No significant change in liver transaminases (in children)  Phase 2: improvement in liver transaminases and fatty liver ultrasonographic findings on mixture of lactobacillus and bifidobacterium species (in children)  Phase 2: improvement in hepatic triglycerides on magnetic resonance spectroscopy and liver transaminases on mixture of lactobacillus and bifidobacterium species  Phase 2: improvement in liver transaminases on mixture of lactobacillus and bifidobacterium species  Pilot study: improvement in liver transaminases with L Bulgaricus and S Thermophilus  Phase 1&2 trials terminated due to no evidence probiotic would benefit the patient | No | [(Alisi et al., 2014; Aller et al., 2011; Famouri et al., 2017; Nabavi et al., 2014; Wong et al., 2013)](https://paperpile.com/c/Q7lYca/jyjM+f1bU+Jn3E+AdHN+PNSw)  <https://clinicaltrials.gov/ct2/show/NCT04074889>  https://clinicaltrials.gov/ct2/show/NCT03511365 |
| **SCD-1 inhibitor** | SCD1 converts saturated fatty acids to monounsaturated fatty acids. SCD1 deficiency has been demonstrated to prevent liver steatosis in several mouse models of NAFLD. Aramchol is an SCD-1 inhibitor. | Phase 2: improvement in hepatic fat content on magnetic resonance spectroscopy in a dose-dependent manner on aramchol  Phase 3 trial in progress | No | [(Safadi et al., 2014)](https://paperpile.com/c/Q7lYca/TkCs)  https://clinicaltrials.gov/ct2/show/NCT04104321 |
| **SGLT2 inhibitor** | SGLT2 inhibitors decrease reabsorption of filtered glucose into the bloodstream via SGLT2 transporter proteins in the kidneys, thus reducing hyperglycaemia. | Phase 4: improvement in hepatic fat content on magnetic resonance spectroscopy on empagliflozin  Phase 2: improvement in hepatic fat content assessed by MRI-PDFF on combination therapy of dapagliflozin and omega-3 fatty acids. Dapagliflozin monotherapy did not reduce hepatic fat but reduced hepatocyte injury biomarkers  Phase 4: reduction in hepatic fat on MRI-PDFF on dapagliflozin | Yes | [(Eriksson et al., 2018; Kahl et al., 2020; Latva-Rasku et al., 2019)](https://paperpile.com/c/Q7lYca/3U2U+xV8H+fI7k) |
| **Silymarin** | Silymarin is a botanical product extracted from milk thistle. It is commonly used as an over the counter supplement in chronic liver diseases. | Phase 2: no improvement in overall NAS. Study limited by substantial number of patients who entered without meeting histological criteria  Phase 2: no significant improvement in overall NAS. Improvement in liver fibrosis | No | [(Navarro et al., 2019; Wah Kheong et al., 2017)](https://paperpile.com/c/Q7lYca/uTM7+MySI) |
| **Statin** | Statins are a widely prescribed class of drugs used to lower cholesterol levels. Their mode of action is primarily via inhibition of HMG-CoA (hydroxymethylglutaryl-coenzyme A) reductase, the rate-limiting enzyme in the cholesterol biosynthesis pathway. They are used routinely in treatment of hyperlipidaemia and cardiovascular disease. | Phase 2: no improvement in hepatic fat assessed by magnetic resonance spectroscopy on pitavastatin  Pilot study: improvement in NASH histology and biochemical markers on rosuvastatin. No placebo included in this study.  Pilot study: no improvement in NASH histology or liver transaminases on simvastatin | No | [(Braun et al., 2018; Kargiotis et al., 2015; Nelson et al., 2009)](https://paperpile.com/c/Q7lYca/L7Ph+oxUa+vaVx) |
| **Thiazolidinediones** | Thiazolidinediones reduce insulin resistance in adipose tissue, muscle and the liver and were routinely used in the management of type 2 diabetes until restrictions were introduced due to safety concerns. They activate PPAR-gamma receptors, thus altering the transcription of several genes involved in glucose and lipid metabolism. They principally act via expanding adipose storage capacity to reduce insulin resistance. | Phase 3: improvement in hepatic steatosis, lobular inflammation and liver transaminases on pioglitazone. No significant improvement in overall NASH or fibrosis  Phase 4: improvement in overall NASH score, fibrosis score and hepatic triglyceride content | No (weight gain) | [(Cusi et al., 2016; Sanyal et al., 2010)](https://paperpile.com/c/Q7lYca/tHJK+KOnO) |
| **UDCA and Tauroursodeoxycholic acid (TUDCA)** | Ursodeoxycholic acid (UDCA) is a natural secondary bile acid. It slows the rate of intestinal cholesterol absorption and facilitates bile flow. It is used in gallstone disease and primary biliary cirrhosis.  TUDCA is a bile acid taurine conjugate derived from ursodeoxycholic acid (UDCA). It is used widely as an over the counter supplement however, unlike UDCA, it is not licenced for medical purposes. | Phase 2: no improvement in overall NAS or biochemical parameters. Improvement in lobular inflammation as a single histological variable  Phase 2: improvement in liver transaminases, metabolic parameters and serum markers of fibrosis  RCT: no improvement in histological or biochemical parameters compared to placebo | No | [(Leuschner et al., 2010; Lindor et al., 2004; Ratziu et al., 2011)](https://paperpile.com/c/Q7lYca/wWB6+gprD+eCd6) |
| **Vitamin D** | Vitamin D is a key regulator in the parathyroid hormone axis and thus in calcium and phosphate homeostasis. The role of vitamin D has been extended to a wide range of disease processes including metabolic conditions. Deficiency is common in patients with NAFLD. | Phase 2: improvement in liver transaminases  Phase 2: no improvement in biochemical parameters  Phase 2: no improvement in hepatic fat fraction assessed by magnetic resonance or in biochemical parameters | No | [(Barchetta et al., 2016; Dabbaghmanesh et al., 2018; Geier et al., 2018)](https://paperpile.com/c/Q7lYca/imt8+RRes+gnO1) |
| **Vitamin E** | Vitamin E is an antioxidant which acts as a free radical scavenger, and is widely used as a vitamin supplement. Oxidative stress has been implicated in NAFLD progression and is a target of treatment approaches. | Phase 3: improvement in overall NAS and hepatocellular ballooning. No significant improvement in individual histological scores for steatosis, inflammation, fibrosis or in biochemical parameters  Phase 2: no significant difference in biochemical parameters of NAFLD between lifestyle interventions + placebo and lifestyle interventions + vitamin E (in children)  Phase 2 trial in progress | No | [(Nobili et al., 2006; Sanyal et al., 2010)](https://paperpile.com/c/Q7lYca/tHJK+Plmw)  https://clinicaltrials.gov/ct2/show/NCT04198805 |

**Supplementary File 1. Narrative summary of evidence in humans for drug classes included in this meta-analysis.**

Descriptions of the principle liver-related findings from randomised controlled trials (RCT) both adults and children with NAFLD with references to completed, published studies or protocols for ongoing trials. A dichotomous assessment of whether the drug is associated with weight loss in humans has been added. ACC, Acetyl-CoA carboxylase; ACE, angiotensin-2 converting enzyme; ALT, alanine aminotransferase; ARB, angiotensin receptor blocker; CCR, chemokine receptor; DHA, Docosahexaenoic acid; DPP4, Dipeptidyl-peptidase 4; EPA, eicosapentaenoic acid; FXR, Farnesoid X receptor; GLP-1, Glucagon-like peptide-1; LXR, Liver X receptor; MRI, magnetic resonance imaging; NAC, N-acetylcysteine; NAS, NAFLD Activity Score; NASH, non-alcoholic steatohepatitis; PDE, Phosphodiesterase; PDFF, proton-density fat fraction; PPAR, Peroxisome proliferator-activated receptor; PUFA; omega-3 polyunsaturated fatty acid; RAAS, renin-angiotensin-aldosterone system; SCD1, Stearoyl–CoA desaturase-1; SGLT2, Sodium-glucose co-transporter-2; TUDCA, Tauroursodeoxycholic acid; and UDCA, Ursodeoxycholic acid.

**Supplementary references:**

[Abdel Monem SM. 2017. Probiotic Therapy in Patients with Nonalcoholic Steatohepatitis in Zagazig University Hospitals. *Euroasian J Hepatogastroenterol* **7**:101–106.](http://paperpile.com/b/Q7lYca/pyg1)

[Alisi A, Bedogni G, Baviera G, Giorgio V, Porro E, Paris C, Giammaria P, Reali L, Anania F, Nobili V. 2014. Randomised clinical trial: The beneficial effects of VSL#3 in obese children with non-alcoholic steatohepatitis. *Aliment Pharmacol Ther* **39**:1276–1285.](http://paperpile.com/b/Q7lYca/jyjM)

[Alkhouri N, Lawitz E, Noureddin M, DeFronzo R, Shulman GI. 2020. GS-0976 (Firsocostat): an investigational liver-directed acetyl-CoA carboxylase (ACC) inhibitor for the treatment of non-alcoholic steatohepatitis (NASH). *Expert Opin Investig Drugs* **29**:135–141.](http://paperpile.com/b/Q7lYca/qsjA)

[Aller R, De Luis DA, Izaola O, Conde R, Gonzalez Sagrado M, Primo D, De La Fuente B, Gonzalez J. 2011. Effect of a probiotic on liver aminotransferases in nonalcoholic fatty liver disease patients: a double blind randomized clinical trial. *Eur Rev Med Pharmacol Sci* **15**:1090–1095.](http://paperpile.com/b/Q7lYca/PNSw)

[Argo CK, Patrie JT, Lackner C, Henry TD, de Lange EE, Weltman AL, Shah NL, Al-Osaimi AM, Pramoonjago P, Jayakumar S, Binder LP, Simmons-Egolf WD, Burks SG, Bao Y, Taylor AG, Rodriguez J, Caldwell SH. 2015. Effects of n-3 fish oil on metabolic and histological parameters in NASH: a double-blind, randomized, placebo-controlled trial. *J Hepatol* **62**:190–197.](http://paperpile.com/b/Q7lYca/zacI)

[Armstrong MJ, Gaunt P, Aithal GP, Barton D, Hull D, Parker R, Hazlehurst JM, Guo K, Abouda G, Aldersley MA, Stocken D, Gough SC, Tomlinson JW, Brown RM, H??bscher SG, Newsome PN. 2015. Liraglutide safety and efficacy in patients with non-alcoholic steatohepatitis (LEAN): A multicentre, double-blind, randomised, placebo-controlled phase 2 study. *The Lancet*. doi:](http://paperpile.com/b/Q7lYca/jAo5)[10.1016/S0140-6736(15)00803-X](http://dx.doi.org/10.1016/S0140-6736(15)00803-X)

[Asghari S, Asghari-Jafarabadi M, Somi M-H, Ghavami S-M, Rafraf M. 2018. Comparison of Calorie-Restricted Diet and Resveratrol Supplementation on Anthropometric Indices, Metabolic Parameters, and Serum Sirtuin-1 Levels in Patients With Nonalcoholic Fatty Liver Disease: A Randomized Controlled Clinical Trial. *J Am Coll Nutr* **37**:223–233.](http://paperpile.com/b/Q7lYca/51V1)

[Barchetta I, Del Ben M, Angelico F, Di Martino M, Fraioli A, La Torre G, Saulle R, Perri L, Morini S, Tiberti C, Bertoccini L, Cimini FA, Panimolle F, Catalano C, Baroni MG, Cavallo MG. 2016. No effects of oral vitamin D supplementation on non-alcoholic fatty liver disease in patients with type 2 diabetes: a randomized, double-blind, placebo-controlled trial. *BMC Med* **14**:92.](http://paperpile.com/b/Q7lYca/imt8)

[Bizino MB, Jazet IM, de Heer P, van Eyk HJ, Dekkers IA, Rensen PCN, Paiman EHM, Lamb HJ, Smit JW. 2020. Placebo-controlled randomised trial with liraglutide on magnetic resonance endpoints in individuals with type 2 diabetes: a pre-specified secondary study on ectopic fat accumulation. *Diabetologia* **63**:65–74.](http://paperpile.com/b/Q7lYca/Tklt)

[Braun LR, Feldpausch MN, Czerwonka N, Weiss J, Branch K, Lee H, Martinez-Salazar EL, Torriani M, Sponseller CA, Grinspoon SK, Stanley TL. 2018. Effects of Pitavastatin on Insulin Sensitivity and Liver Fat: A Randomized Clinical Trial. *J Clin Endocrinol Metab* **103**:4176–4186.](http://paperpile.com/b/Q7lYca/oxUa)

[Chachay VS, Macdonald GA, Martin JH, Whitehead JP, O’Moore-Sullivan TM, Lee P, Franklin M, Klein K, Taylor PJ, Ferguson M, Coombes JS, Thomas GP, Cowin GJ, Kirkpatrick CMJ, Prins JB, Hickman IJ. 2014. Resveratrol does not benefit patients with nonalcoholic fatty liver disease. *Clin Gastroenterol Hepatol* **12**:2092–103.e1–6.](http://paperpile.com/b/Q7lYca/jrb3)

[Cui J, Philo L, Nguyen P, Hofflich H, Hernandez C, Bettencourt R, Richards L, Salotti J, Bhatt A, Hooker J, Haufe W, Hooker C, Brenner DA, Sirlin CB, Loomba R. 2016. Sitagliptin vs. placebo for non-alcoholic fatty liver disease: A randomized controlled trial. *J Hepatol* **65**:369–376.](http://paperpile.com/b/Q7lYca/yNL1)

[Cusi K, Orsak B, Bril F, Lomonaco R, Hecht J, Ortiz-Lopez C, Tio F, Hardies J, Darland C, Musi N, Webb A, Portillo-Sanchez P. 2016. Long-term pioglitazone treatment for patients with nonalcoholic steatohepatitis and prediabetes or type 2 diabetes mellitus a randomized trial. *Ann Intern Med* **165**:305–315.](http://paperpile.com/b/Q7lYca/KOnO)

[Dabbaghmanesh MH, Danafar F, Eshraghian A, Omrani GR. 2018. Vitamin D supplementation for the treatment of non-alcoholic fatty liver disease: A randomized double blind placebo controlled trial. *Diabetes Metab Syndr* **12**:513–517.](http://paperpile.com/b/Q7lYca/RRes)

[El-Haggar SM, Mostafa TM. 2015. Comparative clinical study between the effect of fenofibrate alone and its combination with pentoxifylline on biochemical parameters and liver stiffness in patients with non-alcoholic fatty liver disease. *Hepatol Int* **9**:471–479.](http://paperpile.com/b/Q7lYca/ecZv)

[Eriksson JW, Lundkvist P, Jansson P-A, Johansson L, Kvarnström M, Moris L, Miliotis T, Forsberg G-B, Risérus U, Lind L, Oscarsson J. 2018. Effects of dapagliflozin and n-3 carboxylic acids on non-alcoholic fatty liver disease in people with type 2 diabetes: a double-blind randomised placebo-controlled study. *Diabetologia* **61**:1923–1934.](http://paperpile.com/b/Q7lYca/xV8H)

[Fabbrini E, Mohammed BS, Korenblat KM, Magkos F, McCrea J, Patterson BW, Klein S. 2010. Effect of fenofibrate and niacin on intrahepatic triglyceride content, very low-density lipoprotein kinetics, and insulin action in obese subjects with nonalcoholic fatty liver disease. *J Clin Endocrinol Metab* **95**:2727–2735.](http://paperpile.com/b/Q7lYca/kQ7P)

[Faghihzadeh F, Adibi P, Rafiei R, Hekmatdoost A. 2014. Resveratrol supplementation improves inflammatory biomarkers in patients with nonalcoholic fatty liver disease. *Nutr Res* **34**:837–843.](http://paperpile.com/b/Q7lYca/ZF39)

[Famouri F, Shariat Z, Hashemipour M, Keikha M, Kelishadi R. 2017. Effects of Probiotics on Nonalcoholic Fatty Liver Disease in Obese Children and Adolescents. *J Pediatr Gastroenterol Nutr* **64**:413–417.](http://paperpile.com/b/Q7lYca/f1bU)

[Friedman SL, Ratziu V, Harrison SA, Abdelmalek MF, Aithal GP, Caballeria J, Francque S, Farrell G, Kowdley KV, Craxi A, Simon K, Fischer L, Melchor-Khan L, Vest J, Wiens BL, Vig P, Seyedkazemi S, Goodman Z, Wong VW-S, Loomba R, Tacke F, Sanyal A, Lefebvre E. 2018. A randomized, placebo-controlled trial of cenicriviroc for treatment of nonalcoholic steatohepatitis with fibrosis. *Hepatology* **67**:1754–1767.](http://paperpile.com/b/Q7lYca/w5tl)

[Garcia-Tsao G, Bosch J, Kayali Z, Harrison SA, Abdelmalek MF, Lawitz E, Satapathy SK, Ghabril M, Shiffman ML, Younes ZH, Thuluvath PJ, Berzigotti A, Albillos A, Robinson JM, Hagerty DT, Chan JL, Sanyal AJ, IDN-6556-14 Investigators(‡). 2020. Randomized placebo-controlled trial of emricasan for non-alcoholic steatohepatitis-related cirrhosis with severe portal hypertension. *J Hepatol* **72**:885–895.](http://paperpile.com/b/Q7lYca/OzKz)

[Geier A, Eichinger M, Stirnimann G, Semela D, Tay F, Seifert B, Tschopp O, Bantel H, Jahn D, Marques Maggio E, Saleh L, Bischoff-Ferrari HA, Müllhaupt B, Dufour J-F. 2018. Treatment of non-alcoholic steatohepatitis patients with vitamin D: a double-blinded, randomized, placebo-controlled pilot study. *Scand J Gastroenterol* **53**:1114–1120.](http://paperpile.com/b/Q7lYca/gnO1)

[Hajiaghamohammadi AA, Miroliaee A, Samimi R, Alborzi F, Ziaee A. 2013. A Comparison of Ezetimibe and Acarbose in Decreasing Liver Transaminase in Nonalcoholic Fatty Liver Disease: A Randomized Clinical Trial. *Govaresh* **18**:186–190.](http://paperpile.com/b/Q7lYca/QJJ6)

[Harrison SA, Goodman Z, Jabbar A, Vemulapalli R, Younes ZH, Freilich B, Sheikh MY, Schattenberg JM, Kayali Z, Zivony A, Sheikh A, Garcia-Samaniego J, Satapathy SK, Therapondos G, Mena E, Schuppan D, Robinson J, Chan JL, Hagerty DT, Sanyal AJ. 2020. A randomized, placebo-controlled trial of emricasan in patients with NASH and F1-F3 fibrosis. *J Hepatol* **72**:816–827.](http://paperpile.com/b/Q7lYca/TmW9)

[Haukeland JW, Konopski Z, Eggesbø HB, von Volkmann HL, Raschpichler G, Bjøro K, Haaland T, Løberg EM, Birkeland K. 2009. Metformin in patients with non-alcoholic fatty liver disease: a randomized, controlled trial. *Scand J Gastroenterol* **44**:853–860.](http://paperpile.com/b/Q7lYca/dsRg)

[Kahl S, Gancheva S, Straßburger K, Herder C, Machann J, Katsuyama H, Kabisch S, Henkel E, Kopf S, Lagerpusch M, Kantartzis K, Kupriyanova Y, Markgraf D, van Gemert T, Knebel B, Wolkersdorfer MF, Kuss O, Hwang J-H, Bornstein SR, Kasperk C, Stefan N, Pfeiffer A, Birkenfeld AL, Roden M. 2020. Empagliflozin Effectively Lowers Liver Fat Content in Well-Controlled Type 2 Diabetes: A Randomized, Double-Blind, Phase 4, Placebo-Controlled Trial. *Diabetes Care* **43**:298–305.](http://paperpile.com/b/Q7lYca/3U2U)

[Kantartzis K, Fritsche L, Bombrich M, Machann J, Schick F, Staiger H, Kunz I, Schoop R, Lehn-Stefan A, Heni M, Peter A, Fritsche A, Häring H-U, Stefan N. 2018. Effects of resveratrol supplementation on liver fat content in overweight and insulin-resistant subjects: A randomized, double-blind, placebo-controlled clinical trial. *Diabetes Obes Metab* **20**:1793–1797.](http://paperpile.com/b/Q7lYca/xmEs)

[Kargiotis K, Athyros VG, Giouleme O, Katsiki N, Katsiki E, Anagnostis P, Boutari C, Doumas M, Karagiannis A, Mikhailidis DP. 2015. Resolution of non-alcoholic steatohepatitis by rosuvastatin monotherapy in patients with metabolic syndrome. *World J Gastroenterol* **21**:7860–7868.](http://paperpile.com/b/Q7lYca/L7Ph)

[Khoshbaten M, Aliasgarzadeh A, Masnadi K, Tarzamani MK, Farhang S, Babaei H, Kiani J, Zaare M, Najafipoor F. 2010. N-acetylcysteine improves liver function in patients with non-alcoholic Fatty liver disease. *Hepat Mon* **10**:12–16.](http://paperpile.com/b/Q7lYca/GzUI)

[Kim W, Kim BG, Lee JS, Lee CK, Yeon JE, Chang MS, Kim JH, Kim H, Yi S, Lee J, Cho J-Y, Kim SG, Lee J-H, Kim YJ. 2017. Randomised clinical trial: the efficacy and safety of oltipraz, a liver X receptor alpha-inhibitory dithiolethione in patients with non-alcoholic fatty liver disease. *Aliment Pharmacol Ther* **45**:1073–1083.](http://paperpile.com/b/Q7lYca/Qu9m)

[Latva-Rasku A, Honka M-J, Kullberg J, Mononen N, Lehtimäki T, Saltevo J, Kirjavainen AK, Saunavaara V, Iozzo P, Johansson L, Oscarsson J, Hannukainen JC, Nuutila P. 2019. The SGLT2 Inhibitor Dapagliflozin Reduces Liver Fat but Does Not Affect Tissue Insulin Sensitivity: A Randomized, Double-Blind, Placebo-Controlled Study With 8-Week Treatment in Type 2 Diabetes Patients. *Diabetes Care* **42**:931–937.](http://paperpile.com/b/Q7lYca/fI7k)

[Leuschner UFH, Lindenthal B, Herrmann G, Arnold JC, Rössle M, Cordes H-J, Zeuzem S, Hein J, Berg T, NASH Study Group. 2010. High-dose ursodeoxycholic acid therapy for nonalcoholic steatohepatitis: a double-blind, randomized, placebo-controlled trial. *Hepatology* **52**:472–479.](http://paperpile.com/b/Q7lYca/gprD)

[Lindor KD, Kowdley KV, Heathcote EJ, Harrison ME, Jorgensen R, Angulo P, Lymp JF, Burgart L, Colin P. 2004. Ursodeoxycholic Acid for Treatment of Nonalcoholic Steatohepatitis: Results of a Randomized Trial. *Hepatology* **39**:770–778.](http://paperpile.com/b/Q7lYca/wWB6)

[Loomba R, Lutchman G, Kleiner DE, Ricks M, Feld JJ, Borg BB, Modi A, Nagabhyru P, Sumner AE, Liang TJ, Hoofnagle JH. 2009. Clinical trial: pilot study of metformin for the treatment of non-alcoholic steatohepatitis. *Aliment Pharmacol Ther* **29**:172–182.](http://paperpile.com/b/Q7lYca/iPW1)

[Loomba R, Sirlin CB, Ang B, Bettencourt R, Jain R, Salotti J, Soaft L, Hooker J, Kono Y, Bhatt A, Hernandez L, Nguyen P, Noureddin M, Haufe W, Hooker C, Yin M, Ehman R, Lin GY, Valasek MA, Brenner DA, Richards L, San Diego Integrated NAFLD Research Consortium (SINC). 2015. Ezetimibe for the treatment of nonalcoholic steatohepatitis: assessment by novel magnetic resonance imaging and magnetic resonance elastography in a randomized trial (MOZART trial). *Hepatology* **61**:1239–1250.](http://paperpile.com/b/Q7lYca/bEHQ)

[Macauley M, Hollingsworth KG, Smith FE, Thelwall PE, Al-Mrabeh A, Schweizer A, Foley JE, Taylor R. 2015. Effect of vildagliptin on hepatic steatosis. *J Clin Endocrinol Metab* **100**:1578–1585.](http://paperpile.com/b/Q7lYca/28mR)

[McPherson S, Wilkinson N, Tiniakos D, Wilkinson J, Burt AD, McColl E, Stocken DD, Steen N, Barnes J, Goudie N, Stewart S, Bury Y, Mann D, Anstee QM, Day CP. 2017. A randomised controlled trial of losartan as an anti-fibrotic agent in non-alcoholic steatohepatitis. *PLoS One* **12**:1–17.](http://paperpile.com/b/Q7lYca/SSxV)

[Mudaliar S, Henry RR, Sanyal AJ, Morrow L, Marschall H-U, Kipnes M, Adorini L, Sciacca CI, Clopton P, Castelloe E, Dillon P, Pruzanski M, Shapiro D. 2013. Efficacy and safety of the farnesoid X receptor agonist obeticholic acid in patients with type 2 diabetes and nonalcoholic fatty liver disease. *Gastroenterology* **145**:574–82.e1.](http://paperpile.com/b/Q7lYca/1bX7)

[Nabavi S, Rafraf M, Somi MH, Homayouni-Rad A, Asghari-Jafarabadi M. 2014. Effects of probiotic yogurt consumption on metabolic factors in individuals with nonalcoholic fatty liver disease. *J Dairy Sci* **97**:7386–7393.](http://paperpile.com/b/Q7lYca/AdHN)

[Navarro VJ, Belle SH, D’Amato M, Adfhal N, Brunt EM, Fried MW, Reddy KR, Wahed AS, Harrison S, Silymarin in NASH and C Hepatitis (SyNCH) Study Group. 2019. Silymarin in non-cirrhotics with non-alcoholic steatohepatitis: A randomized, double-blind, placebo controlled trial. *PLoS One* **14**:e0221683.](http://paperpile.com/b/Q7lYca/uTM7)

[Nelson A, Torres DM, Morgan AE, Fincke C, Harrison SA. 2009. A pilot study using simvastatin in the treatment of nonalcoholic steatohepatitis: A randomized placebo-controlled trial. *J Clin Gastroenterol* **43**:990–994.](http://paperpile.com/b/Q7lYca/vaVx)

[Neuschwander-Tetri BA, Loomba R, Sanyal AJ, Lavine JE, Van Natta ML, Abdelmalek MF, Chalasani N, Dasarathy S, Diehl AM, Hameed B, Kowdley KV, McCullough A, Terrault N, Clark JM, Tonascia J, Brunt EM, Kleiner DE, Doo E. 2015. Farnesoid X nuclear receptor ligand obeticholic acid for non-cirrhotic, non-alcoholic steatohepatitis (FLINT): A multicentre, randomised, placebo-controlled trial. *Lancet* **385**:956–965.](http://paperpile.com/b/Q7lYca/RlX0)

[Nobili V, Bedogni G, Alisi A, Pietrobattista A, Rise P, Galli C, Agostoni C. 2011. Docosahexaenoic acid supplementation decreases liver fat content in children with non-alcoholic fatty liver disease: double-blind randomised controlled clinical trial. *Arch Dis Child* **96**:350–353.](http://paperpile.com/b/Q7lYca/Vyxp)

[Nobili V, Manco M, Devito R, Ciampalini P, Piemonte F, Marcellini M. 2006. Effect of vitamin E on aminotransferase levels and insulin resistance in children with non-alcoholic fatty liver disease. *Aliment Pharmacol Ther* **24**:1553–1561.](http://paperpile.com/b/Q7lYca/Plmw)

[Nogueira MA, Oliveira CP, Ferreira Alves VA, Stefano JT, Rodrigues LSDR, Torrinhas RS, Cogliati B, Barbeiro H, Carrilho FJ, Waitzberg DL. 2016. Omega-3 polyunsaturated fatty acids in treating non-alcoholic steatohepatitis: A randomized, double-blind, placebo-controlled trial. *Clin Nutr* **35**:578–586.](http://paperpile.com/b/Q7lYca/tGrc)

[Oscarsson J, Önnerhag K, Risérus U, Sundén M, Johansson L, Jansson P-A, Moris L, Nilsson PM, Eriksson JW, Lind L. 2018. Effects of free omega-3 carboxylic acids and fenofibrate on liver fat content in patients with hypertriglyceridemia and non-alcoholic fatty liver disease: A double-blind, randomized, placebo-controlled study. *J Clin Lipidol* **12**:1390–1403.e4.](http://paperpile.com/b/Q7lYca/5zpz)

[Panahi Y, Kianpour P, Mohtashami R, Jafari R, Simental-Mendía LE, Sahebkar A. 2017. Efficacy and Safety of Phytosomal Curcumin in Non-Alcoholic Fatty Liver Disease: A Randomized Controlled Trial. *Drug Res*  **67**:244–251.](http://paperpile.com/b/Q7lYca/m6nt)

[Rahmani S, Asgary S, Askari G, Keshvari M, Hatamipour M, Feizi A, Sahebkar A. 2016. Treatment of Non-alcoholic Fatty Liver Disease with Curcumin: A Randomized Placebo-controlled Trial. *Phytother Res* **30**:1540–1548.](http://paperpile.com/b/Q7lYca/DHwC)

[Ratziu V, de Ledinghen V, Oberti F, Mathurin P, Wartelle-Bladou C, Renou C, Sogni P, Maynard M, Larrey D, Serfaty L, Bonnefont-Rousselot D, Bastard J-P, Rivière M, Spénard J, FRESGUN. 2011. A randomized controlled trial of high-dose ursodesoxycholic acid for nonalcoholic steatohepatitis. *J Hepatol* **54**:1011–1019.](http://paperpile.com/b/Q7lYca/eCd6)

[Ratziu V, Harrison SA, Francque S, Bedossa P, Lehert P, Serfaty L, Romero-Gomez M, Boursier J, Abdelmalek M, Caldwell S, Drenth J, Anstee QM, Hum D, Hanf R, Roudot A, Megnien S, Staels B, Sanyal A, Mathurin P, Gournay J, Nguyen-Khac E, De Ledinghen V, Larrey D, Tran A, Bourliere M, Maynard-Muet M, Asselah T, Henrion J, Nevens F, Cassiman D, Geerts A, Moreno C, Beuers UH, Galle PR, Spengler U, Bugianesi E, Craxi A, Angelico M, Fargion S, Voiculescu M, Gheorghe L, Preotescu L, Caballeria J, Andrade RJ, Crespo J, Callera JL, Ala A, Aithal G, Abouda G, Luketic V, Huang MA, Gordon S, Pockros P, Poordad F, Shores N, Moehlen MW, Bambha K, Clark V, Satapathy S, Parekh S, Reddy RK, Sheikh MY, Szabo G, Vierling J, Foster T, Umpierrez G, Chang C, Box T, Gallegos-Orozco J. 2016. Elafibranor, an Agonist of the Peroxisome Proliferator-Activated Receptor-α and -δ, Induces Resolution of Nonalcoholic Steatohepatitis Without Fibrosis Worsening. *Gastroenterology* **150**:1147–1159e5.](http://paperpile.com/b/Q7lYca/bPuh)

[Safadi R, Konikoff FM, Mahamid M, Zelber-Sagi S, Halpern M, Gilat T, Oren R, FLORA Group. 2014. The fatty acid-bile acid conjugate Aramchol reduces liver fat content in patients with nonalcoholic fatty liver disease. *Clin Gastroenterol Hepatol* **12**:2085–91.e1.](http://paperpile.com/b/Q7lYca/TkCs)

[Sanyal AJ, Abdelmalek MF, Suzuki A, Cummings OW, Chojkier M, EPE-A Study Group. 2014. No significant effects of ethyl-eicosapentanoic acid on histologic features of nonalcoholic steatohepatitis in a phase 2 trial. *Gastroenterology* **147**:377–84.e1.](http://paperpile.com/b/Q7lYca/FCZH)

[Sanyal AJ, Chalasani N, Kowdley KV, McCullough A, Diehl AM, Bass NM, Neuschwander-Tetri BA, Lavine JE, Tonascia J, Unalp A, Van Natta M, Clark J, Brunt EM, Kleiner DE, Hoofnagle JH, Robuck PR. 2010. Pioglitazone, vitamin E, or placebo for nonalcoholic steatohepatitis. *N Engl J Med* **362**:1675–1685.](http://paperpile.com/b/Q7lYca/tHJK)

[Scorletti E, Afolabi PR, Miles EA, Smith DE, Almehmadi A, Alshathry A, Moyses HE, Clough GF, Wright M, Patel J, Bindels L, Delzenne NM, Calder PC, Byrne CD. 2018. Design and rationale of the INSYTE study: A randomised, placebo controlled study to test the efficacy of a synbiotic on liver fat, disease biomarkers and intestinal microbiota in non-alcoholic fatty liver disease. *Contemp Clin Trials* **71**:113–123.](http://paperpile.com/b/Q7lYca/B5UE)

[Scorletti E, Bhatia L, McCormick KG, Clough GF, Nash K, Hodson L, Moyses HE, Calder PC, Byrne CD, WELCOME Study. 2014. Effects of purified eicosapentaenoic and docosahexaenoic acids in nonalcoholic fatty liver disease: results from the Welcome* study. *Hepatology* **60**:1211–1221.](http://paperpile.com/b/Q7lYca/TvhF)

[Shiffman M, Freilich B, Vuppalanchi R, Watt K, Chan JL, Spada A, Hagerty DT, Schiff E. 2019. Randomised clinical trial: emricasan versus placebo significantly decreases ALT and caspase 3/7 activation in subjects with non-alcoholic fatty liver disease. *Aliment Pharmacol Ther* **49**:64–73.](http://paperpile.com/b/Q7lYca/Ovtf)

[Tenorio-Jiménez C, Martínez-Ramírez MJ, Tercero-Lozano M, Arraiza-Irigoyen C, Del Castillo-Codes I, Olza J, Plaza-Díaz J, Fontana L, Migueles JH, Olivares M, Gil Á, Gomez-Llorente C. 2018. Evaluation of the effect of Lactobacillus reuteri V3401 on biomarkers of inflammation, cardiovascular risk and liver steatosis in obese adults with metabolic syndrome: a randomized clinical trial (PROSIR). *BMC Complement Altern Med* **18**:306.](http://paperpile.com/b/Q7lYca/YHah)

[Vajro P, Mandato C, Licenziati MR, Franzese A, Vitale DF, Lenta S, Caropreso M, Vallone G, Meli R. 2011. Effects of Lactobacillus rhamnosus strain GG in pediatric obesity-related liver disease. *J Pediatr Gastroenterol Nutr* **52**:740–743.](http://paperpile.com/b/Q7lYca/lOG0)

[Van Wagner LB, Koppe SWP, Brunt EM, Gottstein J, Gardikiotes K, Green RM, Rinella ME. 2011. Pentoxifylline for the treatment of non-alcoholic steatohepatitis: a randomized controlled trial. *Ann Hepatol* **10**:277–286.](http://paperpile.com/b/Q7lYca/mQ8m)

[Wah Kheong C, Nik Mustapha NR, Mahadeva S. 2017. A Randomized Trial of Silymarin for the Treatment of Nonalcoholic Steatohepatitis. *Clin Gastroenterol Hepatol* **15**:1940–1949.e8.](http://paperpile.com/b/Q7lYca/MySI)

[Wong VW-S, Won GL-H, Chim AM-L, Chu WC-W, Yeung DK-W, Li KC-T, Chan HL-Y. 2013. Treatment of nonalcoholic steatohepatitis with probiotics. A proof-of-concept study. *Ann Hepatol* **12**:256–262.](http://paperpile.com/b/Q7lYca/Jn3E)

[Yaghoubi M, Jafari S, Sajedi B, Gohari S, Akbarieh S, Heydari AH, Jameshoorani M. 2017. Comparison of fenofibrate and pioglitazone effects on patients with nonalcoholic fatty liver disease. *Eur J Gastroenterol Hepatol* **29**:1385–1388.](http://paperpile.com/b/Q7lYca/whAt)

[Yan H-M, Xia M-F, Wang Y, Chang X-X, Yao X-Z, Rao S-X, Zeng M-S, Tu Y-F, Feng R, Jia W-P, Liu J, Deng W, Jiang J-D, Gao X. 2015. Efficacy of Berberine in Patients with Non-Alcoholic Fatty Liver Disease. *PLoS One* **10**:e0134172.](http://paperpile.com/b/Q7lYca/0lCh)

[Zein CO, Yerian LM, Gogate P, Lopez R, Kirwan JP, Feldstein AE, McCullough AJ. 2011. Pentoxifylline improves nonalcoholic steatohepatitis: a randomized placebo-controlled trial. *Hepatology* **54**:1610–1619.](http://paperpile.com/b/Q7lYca/gTiF)
