# Supplementary material for: Weight loss, insulin resistance, and study design confound results in a meta-analysis of animal models of fatty liver
Source: eLife. 2020 Oct 16;9:e56573. doi: 10.7554/eLife.56573 (PMC7647398; doi:10.7554/eLife.56573)
Supplement: Supplementary file 2. — Prospectively registered on SyRF in August 2017. [file elife-56573-supp2.pdf]

Review Title

Systematic review of rodent models of non-alcoholic fatty liver disease: correlation with human phenotype, efficacy of trialled therapeutic interventions and identification of drug targets.

Organisational affiliation of the review

Department of Paediatrics, University of Cambridge
Addenbrooke's Hospital, Cambridge

Review team members and their organisational affiliations

Dr Jake P. Mann, Department of Paediatrics, University of Cambridge
Amedine Duret, University of Cambridge
Dana de Gracia Hahn, University of Cambridge
Harriet Hunter, University of Cambridge
Yu Ri Im, University of Cambridge

Funding sources/sponsors

None

Conflicts of interest

None

Collaborators

Dr. Matthew J. Armstrong, Liver Unit, University Hospital Birmingham

Review question(s)

1. Rodent models of NAFLD: which ones correlate most closely to the phenotype and histology of human NAFLD?
2. Which pharmacological therapy has shown greatest efficacy in animal models?
3. Is there any evidence of overuse of animals?
4. Can potential drug targets can be identified from existing animal model data?

Searches

The following 2 electronic databases will be searched: PubMed via Medline and Embase. The authors will use keywords to screen for studies relating to NAFLD and the use of animal models in this field.

The following search term will be used:
("Non-alcoholic fatty liver disease" OR "Nonalcoholic fatty liver disease" OR "NAFLD" OR "non-alcoholic steatohepatitis" OR "nonalcoholic steatohepatitis" OR "NASH" OR "fatty liver" OR "hepatic steatosis") AND ("mouse" OR "animal" OR "rat" OR "murine" OR "animal model" OR "murine model" OR "rodent model" OR "experimental model") NOT ("Review")

Animal filters will be used for both PubMed and Embase.

Condition or domain being studied

Non-alcoholic fatty liver disease (NAFLD) refers to a spectrum of pathological changes in the the liver characterised by steatosis in >5% of hepatocytes in individuals without excess alcohol consumption. It is commonly considered to be the hepatic manifestation of the metabolic syndrome. There are currently no specific therapeutic interventions for NAFLD available clinically. Multiple animal models have been developed for NAFLD, including those that reflect non-alcoholic fatty liver (NAFL, or “simple steatosis), nonalcoholic steatohepatitis (NASH), fibrosis and hepatocellular carcinoma. They have been used to trial potential drug therapies, but no attempt has yet been made to systematically review the adequacy of these models, the effectiveness of the drugs, and whether pooling data can be used to identify novel drug targets.

Participants/population

Inclusion criteria: we will consider experimental rodent models of nonalcoholic fatty liver disease in humans.

Exclusion criteria: we will not consider experimental models that are not animal models, or animal models of hepatic pathologies other than NASH or NAFLD, and non-rodent animal models.

Intervention(s), exposure(s)

With regards to the second research question, we will consider only interventions that have a therapeutic intent, as opposed to interventions aimed to demonstrate the pathological mechanism of NAFLD. The interventions under consideration will be therapeutic drugs that previously have been or currently are being trialled in humans at Phase II or Phase III.

Comparator(s)/control(s)

No control

Types of study to be included

Inclusion:

Primary research articles which use an rodent model to attempt to mimic human disease.

Exclusion:

Not concerning NAFLD or NASH, not using animal models, reviews, comments, letters, editorials, meta-analyses, ideas, articles not in English (unless there is an available translation).

Primary outcome(s)

Therapeutic intervention that is most effective at treating fibrosis in rodent models of NAFLD

Secondary outcomes

Specific changes in histology (including development of hepatocellular carcinoma), specific changes in phenotype (e.g. insulin resistance, obesity), intermodel variation in each change in histology and phenotype, correlation of animal model data with human therapeutic trials of NAFLD.

Data extraction and coding

Procedure for selecting studies:

Search both databases, combine results and eliminate duplicates.

Screening:

Abstracts and titles will be reviewed to identify relevant studies. A “test sample” of articles will be screened to ensure consistency between reviewers prior to beginning screening. This will be repeated at the full text review stage. Discrepancies will be settled with JM.

Full text review:

Potentially relevant studies will have their full text extracted and reviewers will independently assess articles for inclusion/exclusion criteria. Two reviewers will assess each article. Any discrepancies will be settled by discussion with JM.

Procedure for data extraction:

A data collection form will be constructed to facilitate data extraction. Data will be extracted in duplicate and assessed for consistency between reviewers. Discrepancies will be resolved by discussion with JM prior to consensus data being used in analysis.

Data extracted will include:

- Paper characteristics: Title, journal, year of publication
- Study details: year of study, location
- Characteristics of animal model used
- Histological comparison to human NAFLD to assess suitability of model
- Drug treatment(s) trialled on animal model, if done
- Outcomes of treatment(s) and histological and phenotypical changes after treatment

Risk of bias (quality) assessment

We will assess risk of bias using sample size calculation, randomisation and blinding. Publication bias will also be assessed using funnel plots.

Strategy for data synthesis

Histological outcomes will be meta-analysed, based on the type of animal model under consideration and the therapeutic used. Model phenotypes will be described through qualitative data synthesis.

Analysis of subgroups or subsets

We will analyse outcomes by animal or by model.

Dissemination plan

Publication in a relevant specialty-specific journal.

Anticipated start date

21/08/2017

Current stage:

screening of potentially relevant abstracts

Anticipated completion date

21/05/2018

Contact details for further information

Jake Mann
jm2032@cam.ac.uk

Language

English

Country

UK
