# Supplementary material for: Weight loss, insulin resistance, and study design confound results in a meta-analysis of animal models of fatty liver
Source: eLife. 2020 Oct 16;9:e56573. doi: 10.7554/eLife.56573 (PMC7647398; doi:10.7554/eLife.56573)
Supplement: Peer review history

# Decision letter

**DOI:** 10.7554/eLife.56573.sa1

**Affiliations:** University of Texas Southwestern Medical CenterUnited States; The Berlin Institute of HealthGermany

In the interests of transparency, eLife publishes the most substantive revision requests and the accompanying author responses.

**Acceptance summary:**

Your systematic analysis of animal studies of fatty liver disease as they compare to human disease is timely. In particular, your studies will be of interest to colleagues in the pharmaceutical industry who are working to develop treatments for the growing problem of NAFLD.

**Decision letter after peer review:**

Thank you for submitting your article "Multiple drug classes show similar treatment effect sizes in animal models of fatty liver disease" for consideration by *eLife*. Your article has been reviewed by two peer reviewers, and the evaluation has been overseen by a Reviewing Editor and Eduardo Franco as the Senior Editor. The following individual involved in review of your submission has agreed to reveal their identity: Sarah McCann (Reviewer #2).

The reviewers have discussed the reviews with one another and the Reviewing Editor has drafted this decision to help you prepare a revised submission.

As the editors have judged that your manuscript is of interest, but as described below that additional experiments are required before it is published, we would like to draw your attention to changes in our revision policy that we have made in response to COVID-19 (https://elifesciences.org/articles/57162). First, because many researchers have temporarily lost access to the labs, we will give authors as much time as they need to submit revised manuscripts. We are also offering, if you choose, to post the manuscript to bioRxiv (if it is not already there) along with this decision letter and a formal designation that the manuscript is "in revision at *eLife*". Please let us know if you would like to pursue this option. (If your work is more suitable for medRxiv, you will need to post the preprint yourself, as the mechanisms for us to do so are still in development.)

Summary:

This study provides a comprehensive overview of the field of preclinical NAFLD research. These reviews are important for summarizing and evaluating current evidence and identifying gaps and areas for improvement for future research. It is clearly structured and follows widely accepted methods. The lack of a pre-specified statistical analysis plan and deviations from the protocol represent possible risks of bias in the review and the search is out of date. I'm unsure whether the relatively strong emphasis on the weak association between drug class and treatment effect is justified. Importantly, the data are discussed in relation to current issues in preclinical research around reproducibility and translation.

Essential revisions:

A very major concern is the overly broad inclusion and grouping criteria used to structure this analysis. It seems to be too broad for a biologically meaningful and productive meta analysis. NAFLD is a heterogenous mix of states (steatosis, steatitis, cirrhosis). Liver triglyceride per se is not a robust measure of NAFLD. A reduction in liver triglyceride alone would likely not be sufficient for regulatory approval for a NAFLD drug. Were the studies that were pooled looking at liver triglyceride as their primary metric, or were they a mix, including other (inflammation, fibrosis) end points more relevant to NAFLD?

How were the drug classes chosen? There seem to be four types of classes, which are not equally valid groupings. 1) Some drug classes include only are a single compound (e.g. Vit E, curcumin,.…), 2) others are groups with a common primary mechanism (e.g. DPP4 inhibitor, SGLT2 inhibitor, FXR agonist, etc), but include different compounds so off-target activities presumably vary, 3) others are poorly defined entities that one could argue should constituent each be treated as separate single compounds (e.g. probiotics, Protoberberine alkaloids, polyphenols), and 4) well defined classes which can have individual members with very different biological activities (e.g., bile acids, ), again, suggesting the more subgroups might be needed for biological sense.

A major determinant of liver triglyceride is body triglyceride. Why was body weight not included in the analysis? I do not think using % overcomes the massive differences in liver triglyceride due to massive differences in body weight.

'Model' includes both genetic models and diet models, finding only 10 categories. This seems like an overly small number of categories. There are many kinds of high-fat diets that have very different compositions and that give very different results.

The analyte in this study is % loss of liver triglyceride. How robust a metric is this for a meta analysis? Liver triglyceride correlates with total body triglyceride and body weight; loss of enough body weight will cause an experiment to be stopped for humane reasons. So the % liver triglyceride would seem to me to be a potentially truncated/limited/bounded metric. Is this affecting the variation that is needed/used for the meta analysis?

It is great to see that there was a published study protocol, however there was no statistical analysis plan specified. There are also several deviations from the protocol that have not been addressed e.g. lack of secondary outcomes, not addressing key research questions as planned, analysis of subgroups. The search is 2.5 years old and while there are over 200 included studies, representing a substantial body of work, the authors note this is a highly active field and thus it is could be a substantial limitation if the data are out of date.

It is reported that there is weak evidence of difference between drug classes and p values are given (p=0.014 subgroup analysis; p=0.002 meta-reg) but as there is no pre-specified statistical analysis plan and no description in the Materials and methods, we don't know what the authors consider to be significant effects. The authors conclude quite strongly that there is limited difference between classes and describe overlapping CIs but e.g. the mean effect of the most effective class is ~4 times higher than the least effective, with no overlapping CIs. It would be useful to know when interpreting these results what a clinically relevant treatment effect would be. The wide CIs limit strong conclusions but as the authors note, the number of animals in each group is low and some classes have few comparisons.

I found the description that "The confidence intervals of 20 out of 21 drug classes overlapped" slightly confusing (Figure 2) when Thiazolidinediones CIs do not overlap with at least 3 other classes but do overlap with many others.

There is also substantial within-class heterogeneity for some drug classes. Could there be differences in efficacy between individual drugs not captured at the class level? How many individual drugs are represented by each class? (Post hoc) subgroup analysis of classes with sufficient comparisons might provide more nuanced information on treatment response and factors affecting efficacy.

While it is dangerous to over-interpret results, I would probably recommend softening the argument of limited differences slightly.

Does it make sense to look at drug dose as a potential source of heterogeneity across different drugs and classes? We would most likely expect individual drugs to have different effective doses.

The multivariable regression model explains ~50% of the heterogeneity in a subset of the studies but is not discussed anywhere in the manuscript (10 variables/71 studies?) Is there something about these studies that can provide information on the broader dataset or is this analysis of limited value?

As only drugs/classes that have been used in clinical trials have been included, it would be interesting to note any similarities/differences in results e.g. GLP-1 agonists demonstrate a robust effect in the current data and the authors report that they demonstrated potential efficacy in clinical trial. Is there anything we can learn from examples of potentially successful translation?

# Reply: Author response

**DOI:** 10.7554/eLife.56573.sa2

Essential revisions:

A very major concern is the overly broad inclusion and grouping criteria used to structure this analysis. It seems to be too broad for a biologically meaningful and productive meta analysis. NAFLD is a heterogenous mix of states (steatosis, steatitis, cirrhosis). Liver triglyceride per se is not a robust measure of NAFLD. A reduction in liver triglyceride alone would likely not be sufficient for regulatory approval for a NAFLD drug. Were the studies that were pooled looking at liver triglyceride as their primary metric, or were they a mix, including other (inflammation, fibrosis) end points more relevant to NAFLD?

Thank you for this suggestion. We agree that liver triglyceride content, particularly in isolation, is not the optimum indicator of NAFLD severity. Therefore we have now added meta-analyses for histological outcomes: steatosis, lobular inflammation, ballooning, overall NAFLD Activity Score, and fibrosis stage. (We also collected data on portal inflammation severity but there was insufficient data for meta-analysis.) We consider this now to appropriately reflect the spectrum of disease in NAFLD.

The included studies rarely identified a primary outcome and they were difficult to categorise into, for example, those focusing on a ‘liver phenotype’ versus those focusing on the systemic ‘metabolic syndrome’. Therefore we believe that reporting both hepatic TG content as well as histology is the least biased method.

Reassuringly, we observed similar trends for multiple outcomes. We have focused the main conclusions of our study on those that were consistent across several meta-analyses and meta-regressions.

Though histological outcomes are more directly comparable to human data, we have retained hepatic TG as an outcome because it is the most widely reported measure.

How were the drug classes chosen? There seem to be four types of classes, which are not equally valid groupings. 1) Some drug classes include only are a single compound (e.g. Vit E, curcumin,.…), 2) others are groups with a common primary mechanism (e.g. DPP4 inhibitor, SGLT2 inhibitor, FXR agonist, etc), but include different compounds so off-target activities presumably vary, 3) others are poorly defined entities that one could argue should constituent each be treated as separate single compounds (e.g. probiotics, Protoberberine alkaloids, polyphenols), and 4) well defined classes which can have individual members with very different biological activities (e.g., bile acids, ), again, suggesting the more subgroups might be needed for biological sense.

We appreciate that the grouping of drugs into classes is a simplification and it was challenging to be consistent as not all the drugs neatly fit into pharmacological classes. We had originally aimed to group drugs based on their mechanism of action. We have now refined the grouping, being more stringent to separate drugs where possible and where their mechanism is less well established. We have added detail to the Materials and methods to describe the process of grouping drugs into classes.

We have also run each meta-analysis with sub-grouping by individual drugs and see consistent trends for the principal findings.

We appreciate that there are some drug groups that remain broad, in particular “Probiotics (mix)” and “Omega-3 polyunsaturated fatty acids (mix)”. Where possible we have separated groups within them, specifically *Lactobacillus* / *Bifidobacterium* and eicosapentaenoic acid / docosahexaenoic acid. However we have retained these broader groups to reduce the number of studies being excluded.

We have also added a section in the limitations of the Discussion to address that this is a simplification and grouping is variably imprecise. We have also specifically highlighted to that conclusions from less well-defined classes may need to be interpreted with caution.

A major determinant of liver triglyceride is body triglyceride. Why was body weight not included in the analysis? I do not think using % overcomes the massive differences in liver triglyceride due to massive differences in body weight.

Thank you for this suggestion and we apologise for not including this initially. Given the body weight often influences liver triglyceride via insulin resistance, we have now performed a series of analyses to address this. We felt it was important to include glucose (and insulin) as there are some models (particularly lipodystrophic models) that may develop marked hepatic steatosis without becoming obese but are insulin resistant.

We have collected data for, and calculated, the difference in body weight, fasting glucose, and fasting insulin for all included studies, then included these as variables in meta-regression. We have found highly consistent associations across multiple outcomes where weight loss and lower insulin/glucose are associated with greater treatment response.

We noted that many drugs were associated significant differences in weight, glucose, or insulin, in animals though they do not cause such responses in humans (e.g. vitamin D or statins). Our interpretation is that the weight loss and change in insulin sensitivity seen in animals may be confounding results and contributing to the lack of translation from animals to humans.

We do appreciate the fasting insulin and glucose are technically not direct measures of insulin resistance, however they are the most widely reported metrics. We have added a section in the limitations to acknowledge that other data (e.g. hyperinsulinaemic-euglycaemic clamps) would be more accurate.

'Model' includes both genetic models and diet models, finding only 10 categories. This seems like an overly small number of categories. There are many kinds of high-fat diets that have very different compositions and that give very different results.

We realise that this is one of several simplifications we have performed in an attempt to synthesise the data. We performed a categorical grouping of models as a ‘high-level’ descriptor. This identified 137 different ‘core’ model categories. They are found in Column E (“Model_simple”) of Figure 1—source data 1 (i.e. the raw data used in the meta-analysis). These are highly variable, for example: “SREBP-1c transgenic overepression + High-fat and high-fructose diet”, “ApoE knockout (ApoE -/-) + high-fat, high-cholesterol diet + T0901317 (LXR agonist)”, and “Pemt-/- + high-fat diet”.

In order for these Models to be included as variables in the meta-regression we required a minimum of 3 unique studies to use them. Due to variation and the high number of studies that used a few common models (e.g. “high-fat diet”, “Leptin deficiency (ob/ob)”), comparatively few different models were included in the meta-regression. We appreciate that our analysis will not have captured the full variation of Model and we have now addressed this as a limitation in the Discussion.

In addition, we have now added data on other dietary constituents (cholesterol, sucrose, and fructose/glucose), which have been included as variables in the meta-regression. Again, we appreciate that this is simplifying the complexity of dietary models however we now use these data to draw general conclusions that diet can affect treatment response. We have specifically commented that analyses of fat %kcal in isolation may be misleading because lower fat %kcal is more frequently coupled with added cholesterol (and/or fructose) than 60% kcal fat diets.

Whilst updating our analyses, we noted that many continuous variables used in the meta-regression were heavily skewed. We have therefore log-transformed them prior to analysis.

The analyte in this study is % loss of liver triglyceride. How robust a metric is this for a meta analysis? Liver triglyceride correlates with total body triglyceride and body weight; loss of enough body weight will cause an experiment to be stopped for humane reasons. So the % liver triglyceride would seem to me to be a potentially truncated/limited/bounded metric. Is this affecting the variation that is needed/used for the meta analysis?

Thank you for highlighting this point. To our knowledge, the most systematic previous analysis of liver fat in animals was from the Hybrid Mouse Diversity Panel (Hui et al., 2015) who found >30 fold variation in hepatic TG across strains.

It is difficult to know whether studies were stopped early due to animal health (including weight loss). We have now included weight difference between placebo and intervention as a variable, which demonstrated a strong association with hepatic TG.

As described above, we have now added multiple histological outcome measures, therefore we have increased confidence in our findings given that we are no longer basing our conclusions on a single metric.

Whilst we did not formally record this, we can anecdotally report that fewer than five of all included studies reported specifics of harm to animals or protocol changes due to animal welfare.

We also note that the range of percentage change in hepatic TG is similar to that reported in another pre-clinical meta-regression analysis in a different field (Watzlawick et al., 2019). Therefore we believe that the dynamic range is appropriate for the analyses performed, especially in combination with the other metrics used.

It is great to see that there was a published study protocol, however there was no statistical analysis plan specified. There are also several deviations from the protocol that have not been addressed e.g. lack of secondary outcomes, not addressing key research questions as planned, analysis of subgroups. The search is 2.5 years old and while there are over 200 included studies, representing a substantial body of work, the authors note this is a highly active field and thus it is could be a substantial limitation if the data are out of date.

We appreciate that the lack of a pre-specified statistical analysis plan is a potential risk of bias. We believe that through sharing our full raw data, code for analysis, the results of all analyses conducted (i.e. not selective reporting) we have minimised the bias introduced from this. We have also specifically commented on it in the Discussion to highlight it as a limitation.

In addition, we have improved the methodology we have used for multivariable meta-analysis to use an approach less open to bias, which considers all variables for importance. We now present results from multi-model inference to determine the top model, which is then subjected to a multiple permutation test to determine the fit of the final models.

With this revised version of the study we have now been able to address several of our secondary outcomes regarding liver histology and insulin resistance/weight.

In the future we hope to build on this initial database to further study our secondary outcomes with a more qualitative approach but we would consider that to be a separate project and anticipate that would take a substantial duration. We trust that the revisions to the current study have made it a more complete analysis to address one research question.

We have now updated our search to January 2019, which required screening a further 1118 articles (991 for full-text review). This, combined with addition of studies reporting histology, has increased the number of included studies from 244 (414 cohorts) to 414 studies (603 cohorts). We appreciate that this remains out of date however our interpretation is that we have now accumulated sufficient evidence to support the general conclusions we have reached, particularly given that we are focusing on observations that were replicated across multiple outcome measures.

It is reported that there is weak evidence of difference between drug classes and p values are given (p=0.014 subgroup analysis; p=0.002 meta-reg) but as there is no pre-specified statistical analysis plan and no description in the Materials and methods, we don't know what the authors consider to be significant effects. The authors conclude quite strongly that there is limited difference between classes and describe overlapping CIs but e.g. the mean effect of the most effective class is ~4 times higher than the least effective, with no overlapping CIs. It would be useful to know when interpreting these results what a clinically relevant treatment effect would be. The wide CIs limit strong conclusions but as the authors note, the number of animals in each group is low and some classes have few comparisons.

We acknowledge that it was not helpful to make non-specific statements about the magnitude of effects, such as ‘weak’ or ‘large’ without pre-specifying criteria. We have added to our Materials and methods to detail how all analyses were assessed for significance.

We have also tempered our discussion regarding the similarity of drug classes. We have commented where confidence intervals have overlapped with the 95% CI for the overall estimate and stated which drugs had deviated from the others based on their 95% CI.

There is a recently established benchmark for change in hepatic TG: a reduction of ≥30% on MRI-measured liver fat is linked to a significant improvement in histology. We have now included this comparison in our manuscript (Jayakumar et al., 2019; Loomba et al., 2020; Stine et al., 2020).

We have also added a table (Supplementary file 1) to provide a narrative summary of the evidence for each drug class studied, with references to the key published RCTs.

I found the description that "The confidence intervals of 20 out of 21 drug classes overlapped" slightly confusing (Figure 2) when Thiazolidinediones CIs do not overlap with at least 3 other classes but do overlap with many others.

We apologise for not being clear. As described above, we have removed non-specific statements about overlapping drug classes and have provided further clarity on the criteria against each analysis was assessed.

There is also substantial within-class heterogeneity for some drug classes. Could there be differences in efficacy between individual drugs not captured at the class level? How many individual drugs are represented by each class? (Post hoc) subgroup analysis of classes with sufficient comparisons might provide more nuanced information on treatment response and factors affecting efficacy.

Thank you for this suggestion. We have now performed a sub-analysis by individual drugs for each outcome metric.

We found that considerable heterogeneity remains even when analysing by individual drug, a trend which was replicated for multiple outcomes.

There is variation of effect size within drug classes where more than one drug contributed (e.g. statin), however it is difficult to make firm conclusion about superiority individual drugs within classes because their confidence intervals overlap. We have provided the full results for all analyses to allow readers to examine results for individual drugs that may be of interest.

While it is dangerous to over-interpret results, I would probably recommend softening the argument of limited differences slightly.

We acknowledge that our original phrasing was too bold. We have edited the language and our conclusions significantly in light of our new results.

Does it make sense to look at drug dose as a potential source of heterogeneity across different drugs and classes? We would most likely expect individual drugs to have different effective doses.

This is an important suggestion and we had also expected to observe an effect of drug dose, however we did not find one in our original submission. We have checked and re-analysed the data to ensure we weren’t missing an effect and excluding any studies that used unusual drug doses or units. We then scaled all drug doses by each individual drug to facilitate maximum power for meta-regression. With this methodology we still did not observe any effect using our standard minimum of 3 studies per drug for inclusion. We also tried re-running the analysis where at least 10 studies were required for each drug and still did not observe any effect.

For example, this bubble plot reflects scaled drug dose (x-axis) and mean difference in hepatic TG (y-axis) for fenofibrate, the drug with the largest treatment effect size and had a suitable number of data points for meta-regression:

**Author response image 1.** 

We interpret this observation to be a further explanation for challenging translation from animals to humans and have added a comment regarding this in the Discussion.

The multivariable regression model explains ~50% of the heterogeneity in a subset of the studies but is not discussed anywhere in the manuscript (10 variables/71 studies?) Is there something about these studies that can provide information on the broader dataset or is this analysis of limited value?

Apologies for not having discussed these results thoroughly. Following the updates we have described above, we have more robust multivariable meta-regression results and improved performance of models. Several models (particularly for NAS and Steatosis grade) account for >60% of variation, without including drug as a variable, and remain robust after a permutation test.

Whilst we could not replicate the same model accuracy across all outcomes, we have used multimodel inference to provide additional evidence for the importance of individual variables (e.g. fat %kcal, glucose difference). We now conclude these analyses illustrate how the combination of study design and weight loss / insulin resistance are central to determining treatment response in NAFLD.

As only drugs/classes that have been used in clinical trials have been included, it would be interesting to note any similarities/differences in results e.g. GLP-1 agonists demonstrate a robust effect in the current data and the authors report that they demonstrated potential efficacy in clinical trial. Is there anything we can learn from examples of potentially successful translation?

This is a key question and we have now attempted to address it in more detail in the Discussion. The drugs that show good efficacy in humans (thiazolidinediones, GLP-1 agonists) do not stand out at the top of any outcome measure. Conversely, fibrates are consistently the drug class with the most efficacy in animals but do not show efficacy in humans.

Unfortunately, our interpretation of these findings is that there is no clearly discernible pattern.
